# Randomized clinical trial to compare the efficacy of ivermectin versus placebo to negativize nasopharyngeal PCR in patients with early COVID-19 in Peru (SAINT-Peru): a structured summary of a study protocol for randomized controlled trial

**DOI:** 10.1186/s13063-021-05236-2

**Published:** 2021-04-09

**Authors:** Patricia J. Garcia, Hansel Mundaca, Cesar Ugarte-Gil, Patricia Leon, German Malaga, Carlos Chaccour, Cesar P. Carcamo

**Affiliations:** 1 School of Public Health and Administration, Universidad Peruana Cayetano Heredia, Lima, Peru; 2 Instituto de Salud Global (ISGlobal), Barcelona, Spain; 3 School of Medicine, Universidad Peruana Cayetano Heredia, Lima, Peru; 4 Tropical Medicine Institute Alexander von Humboldt, Universidad Peruana Cayetano Heredia, Lima, Peru; 5 Hospital Nacional Cayetano Heredia, Lima, Peru; 6 Facultad de Medicina, Universidad de Navarra, Pamplona, Spain

**Keywords:** COVID-19, Randomized controlled trial, protocol, SARS-CoV-2, PCR, early treatment, ivermectin, antiviral, immunomodulatory, Peru

## Abstract

**Objectives:**

The primary objective is to determine the effect of a daily dose of ivermectin administered in three consecutive days to non-severe COVID-19 patients with no more than 96 hours of symptoms, on the detection of SARS-CoV-2 RNA by PCR from nasopharyngeal swabs at day seven post-treatment initiation.

The secondary objectives are:

1. To assess the efficacy of ivermectin to reduce the SARS-CoV-2 viral load in the nasopharyngeal swab on days 4, 7, 14 and 21 post-treatment initiation
2. To assess the efficacy of ivermectin on the improvement of symptoms
3. To assess the proportion of seroconversions at day 21
4. To assess the safety of ivermectin at the proposed dose
5. To determine the magnitude of the immune response against SARS-CoV-2
6. To assess correlation of the presence of intestinal helminths on participants on baseline and day 14 with COVID-19 progression and treatment.

**Trial design:**

SAINT PERU is a triple-blinded, randomized, placebo-controlled trial with two parallel arms to evaluate the efficacy of ivermectin in negativizing nasopharyngeal PCR in patients with SARS-CoV-2 infection.

**Participants:**

The trial is conducted in two national hospitals in Lima-Peru. The study population is patients with a positive PCR test for SARS-CoV-2 in a nasopharyngeal specimen, symptomatic for 96 hours or less, with non-severe COVID-19 disease at baseline, regardless of the presence of risk factors for progression to severity. The study will not include pregnant women or minors (17 years old or younger).

Inclusion criteria

1. COVID-19 symptomatology (cough, fever, anosmia, etc.) lasting no more than 96 hours, with a positive nasopharyngeal swab PCR test for SARS-CoV-2.
2. 18 years or older.
3. No use of ivermectin in the month prior to the visit.
4. No known history of ivermectin allergy.
5. Capable to give informed consent.
6. Not current use of CYP 3A4 or P-gp inhibitor drugs such as quinidine, amiodarone, diltiazem, spironolactone, verapamil, clarithromycin, erythromycin, itraconazole, ketoconazole, cyclosporine, tacrolimus, indinavir, ritonavir, cobicistat or critical CYP3A4 substrate drugs such as warfarin.

Exclusion criteria

1. COVID-19 pneumonia diagnosed by the attending physician (oxygen saturation < 95% or lung examination)
2. Positive pregnancy test for women at childbearing age.
3. Positive IgG against SARS-CoV-2 by rapid diagnostic test at screening.

Participants will be recruited by the investigators at the emergency services of the study sites. They are expected to remain in the trial for a period of 21 days. Follow-up visits will be conducted by the trial medical staff at the participant's home or at a hospital in case of hospitalization. Follow-up visits will assess clinical and laboratory parameters of the patients.

**Intervention and comparator:**

Ivermectin (300 mcg/kg) or placebo will be administered in one daily dose for three consecutive days. Currently, there is no solid data on the efficacy of ivermectin against the virus *in vivo*; therefore the use of placebo in the control group is ethically justified.

**Main outcomes:**

Primary

Proportion of patients with a positive SARS-CoV-2 PCR from a nasopharyngeal swab at day 7 post-treatment.

Secondary

1. Mean viral load as determined by PCR cycle threshold (Ct) on days 4, 7, 14, and 21 2. Proportion of patients with fever and cough at days 4, 7, 14, and 21 as well as proportion of patients progressing to severe disease or death during the trial
2. Proportion of patients with a positive rapid diagnostic test at day 21
3. Proportion of drug-related adverse events during the trial
4. Median levels of IgG, IgM, IgA measured by Luminex

**Randomization:**

Participants will be randomized to receive one dose of 300 mcg/kg ivermectin or placebo daily for three consecutive days. The epidemiologist will generate a list of correlative numbers, in randomized blocks of size 4, with the assignment to the treatment groups (a and b). The randomization list will be kept in an encrypted file accessible only to the trial statistician. This list will be handed directly to the pharmacist. Independently, the principal investigator will randomly assign the intervention (ivermectin) to one of the two groups (a or b) by tossing a coin, and will inform the pharmacist of the result of this process. The pharmacist will prepare and label the treatment vials according to the randomization list prepared by the epidemiologist and the treatment assignment given by the principal investigator. Eligible patients will be allocated in a 1:1 ratio using this randomization list.

**Blinding (masking):**

The clinical trial team, the statistician, and the patients will be blinded as to arm allocation.

The vials with placebo will be visibly identical to the ones with the active drug. Treatment will be administered by staff not involved in the clinical care or participant’s follow up.

**Numbers to be randomized (sample size):**

The planned sample size is 186 SARS-CoV-2 PCR positive patients: 93 patients to treatment and 93 to the placebo group.

**Trial Status:**

Current protocol version: 2.0 dated January 15^th^, 2021. Recruitment started on Aug 29^th^, 2020. Recruitment is expected to be completed April 30^th^ 2021.

**Trial registration:**

“Ensayo Clínico aleatorizado de Fase IIa para comparar la efectividad de la ivermectina versus placebo en la negativización del PCR en pacientes en fase temprana de COVID-19” Peru National Health Institute REPEC with number: PER-034-20, registered July 17^th^ 2020 (National Peruvian Registration before the first participant enrolled).

“Randomized Phase IIA Clinical Trial to Evaluate the Efficacy of Ivermectin to Obtain Negative PCR Results in Patients With Early Phase COVID-19” Clinicaltrials.gov: NCT04635943, retrospectively registered in November 19^th^ 2020

**Full protocol:**

The full protocol is attached as an additional file, accessible from the Trials website (Additional file 1). In the interest of expediting dissemination of this material, the familiar formatting has been eliminated; this Letter serves as a summary of the key elements of the full protocol.

**Supplementary Information:**

The online version contains supplementary material available at 10.1186/s13063-021-05236-2.

## Supplementary Information

**Additional file 1.** Full study protocol.

## Data Availability

The sponsor, the trial sites and study staff will handle the subject’s personal and trial data according to the effective legislation regarding data protection. Collected data will be shared with other ongoing clinical trials on the same topic for individual patient data (IPD) meta analysis or shared upon relevant requests. A de-identified participant-level dataset will be made publicly available not later than six months after trial completion.

