# Supplementary material for: Randomized clinical trial to compare the efficacy of ivermectin versus placebo to negativize nasopharyngeal PCR in patients with early COVID-19 in Peru (SAINT-Peru): a structured summary of a study protocol for randomized controlled trial
Source: Trials. 2021 Apr 9;22:262. doi: 10.1186/s13063-021-05236-2 (PMC8033091; doi:10.1186/s13063-021-05236-2)
Supplement: Supplementary file 1 — Additional file 1. Full study protocol. [file 13063_2021_5236_MOESM1_ESM.pdf]

Título de ensayo: Ensayo Clínico aleatorizado de Fase IIa para comparar la efectividad de la ivermectina versus placebo en la negativización del PCR en pacientes en fase temprana de COVID-19

PROTOCOLO

Investigadores (en orden alfabético):

Investigadora Principal: Patricia García Funegra

Co-Investigadores: Carlos Chaccour

César Cárcamo Cavagnaro

Patricia León

Germán Málaga Rodríguez

Hansel Mundaca Hurtado

César Ugarte Gil

ABREVIATURAS: 5
1. RESUMEN 6
2. ANTECEDENTES Y JUSTIFICIÓN DEL ESTUDIO 7
2.1. Producto en Investigación 9
2.2. Enfermedad bajo estudio 14
2.3. Evaluación de la relación beneficio/riesgo para los participantes en el ensayo clínico 14
3. OBJETIVOS DEL ESTUDIO 15
3.1 Objetivo principal 15
3.2 Objetivos secundarios 15
4. DISEÑO DE ENSAYO 15
4.1. Diseño 16
5. SELECCIÓN DE LOS PACIENTES DE ESTUDIO 19
5.1. Criterios de inclusión 19
5.2. Criterios de exclusión 19
5.3. Criterios para la retirada del estudio 19
6. DESCRIPCIÓN DEL TRATAMIENTO DEL ENSAYO 20
6.1. Método de asignación de tratamiento 20
6.2 Información farmacéutica 20
6.3. Identidad de producto 21
6.4. Riesgos potenciales 22
6.5. Administración 22
6.6. Empaque y rotulado 22
6.7. Manejo y almacenamiento 22
6.8. Dispensación 22
6.9. Responsabilidad 22
6.10. Cumplimiento de intervención de estudio 23
6.11. Eliminación y destrucción 23
6.12. Medicación / dispositivos concomitantes 23
6.13. Escala de dosis 24
6.14. Modificación de dosis y retraso 24
6.15. Toxicidad limitante de dosis 24
6.16. Sobredosis 24
6.17. Interrupción del tratamiento 24
7. EVALUACIÓN DE RESPUESTA 25

7.1. Plan del estudio y procedimientos 25
7.1.1. Obtención del consentimiento 25
7.1.2. Invitación a participar, consentimiento informado, evaluación de elegibilidad y visita basal 25
7.1.3. Visitas subsecuentes de estudio 26
7.2. Variables de valoración 28
8. VALORACIÓN DE SEGURIDAD 28
8.1. Definiciones 29
8.2. Registro y comunicación de eventos adversos 30
8.3. Eventos adversos serios que no requieren comunicación a la persona responsable de farmacovigilancia 31
8.4. Procedimiento de notificación de EAS 31
8.5. Interrupción del producto en investigación debido a eventos adversos 32
9. Análisis estadístico 32
9.1. Descripción de los métodos estadísticos a ser usados 32
EVALUACIÓN DE LA EFICACIA 32
EVALUACIÓN DE SEGURIDAD 32
9.2. Número de sujetos planeados a ser incluidos y poder del estudio 32
9.3. Nivel de significancia a ser usado 33
9.4. Criterios para terminar el estudio 33
9.5. Procedimiento usado para contabilizar los datos perdidos, no utilizados e incorrectos 33
10. ASPECTOS ÉTICOS 34
10.1. Buenas prácticas clínicas 34
10.2. Consideraciones éticas 34
10.3. Información para el paciente y consentimiento informado 34
10.4. Confidencialidad de pacientes 35
10.5. Compensación para el paciente 35
10.6. Póliza de seguros 35
11. GESTIÓN DE DATOS 35
11.1. Formularios para Notificación de Casos (ANEXOS 3 al 8 CRF) 35
11.2. Reporte final y Publicación 36
12. REFERENCIAS 37
ANEXO 1: TABLA DE DOSIFICACIÓN DISCRETA 40
ANEXO 2: TABLA DE EVALUACIÓN DE ELEGIBILIDAD 42
ANEXO 3: FICHA DE VISITA BASAL 45

ANEXO 4: FICHA MEDICACIÓN CONCOMITANTE 48
ANEXO 5: FICHA DE EVENTOS ADVERSOS 49
ANEXO 6: FICHA DE VISITA SEGUIMIENTO 50
ANEXO 7: FICHA DE EVALUACIÓN CLÍNICA DIARIA 52
ANEXO 8: FORMULARIO DE CONSENTIMIENTO INFORMADO PARA PARTICIPAR EN UN ESTUDIO CLÍNICO 54
ANEXO 9: FORMULARIO DE CONSENTIMIENTO INFORMADO AUTORIZACIÓN DE ALMACENAMIENTO PARA USO FUTURO DE MUESTRAS 58

ABREVIATURAS:

Abreviatura	Término
ANM	Autoridad Nacional de Productos Farmacéuticos, Dispositivos Médicos y Productos Sanitarios, denominada anteriormente como DIGEMID.
ANMAT	Administración Nacional de Medicamentos, Alimentos y Tecnología médica
CIOMS	Council for International Organizations of Medical Sciences
CIP	Código de Investigación de Participante
CNTEI-COVID19	Comité Nacional Transitorio de Ética en Investigación para la evaluación y supervisión de los ensayos clínicos de la enfermedad COVID-19
COVID-19	Infección por el nuevo coronavirus 2019 (SARS-CoV-2)
CRF	Formulario para notificación de casos
CU	Ciclo umbral
DENV	Virus dengue
DOT	Tratamiento directamente observado
EA	Evento Adverso
EAS	Evento Adverso Serio
ETDs	Enfermedades Tropicales Desatendidas
FCI	Formulario de Consentimiento Informado
FDA	Federal Drug Administration
GCP	Buenas Prácticas Clínicas (good clinical practice)
HNCH	Hospital Nacional Cayetano Heredia
INS	Instituto Nacional de Salud
IP	Investigador Principal
OGITT	Oficina General de Investigación y Transferencia Tecnológica
PBMC	Célula mononuclear de sangre periférica (peripheric blood mononuclear cell)
PCR	Reacción en cadena de la polimerasa (polymerase chain reaction)
PI	Producto en Investigación
PK	Farmacocinética
PPS	Paciente Perdido en Seguimiento
SOP	Procedimientos operativos estándar (standart operating procedures)
RA	Reacción adversa
RRP	Registros reportados por los pacientes
UPCH	Universidad Peruana Cayetano Heredia
USAR	Reacción adversa seria e inesperada (unexpected serious adverse reactions)
SUSAR	Presuntas reacciones adversas serias e inesperadas (serious and unexpected suspected adverse reactions)

1. RESUMEN

Título del estudio: **SAINT-PERÚ: Ensayo clínico aleatorizado para comparar la efectividad de la ivermectina versus placebo en la negativización del PCR en pacientes en fase temprana de COVID-19**

Introducción

La actual pandemia por COVID-19 ya ha superado los 7.2 millones de casos y 410, 000 muertes en todo el mundo, sin aún existir un tratamiento definitivo para esta enfermedad. Se necesitan urgentemente métodos para controlar el COVID-19. Dentro de los potenciales enfoques a ser considerados están el tratamiento temprano para reducir la progresión a enfermedad grave, el tratamiento para bloquear o disminuir la transmisión con un fármaco que reduzca la excreción viral en el aire, y la profilaxis. Proponemos actuar en pacientes en fase temprana es decir en la primera semana de enfermedad. Estas estrategias aliviarían la presión del COVID-19 sobre el sistema de salud y disminuirían las muertes indirectas por otras enfermedades que requieran cuidados intensivos.

Justificación y Relevancia:

Ivermectina es un fármaco antiparasitario ampliamente utilizado para el tratamiento y control de ciertas enfermedades tropicales desatendidas el cual ha mostrado un excelente perfil de seguridad con más de 2.5 billones de dosis distribuidas en los últimos 30 años. Sin embargo, aún existe data que apoya su uso en COVID-19. En base a evidencia previa, ivermectina podría tener actividad contra COVID-19 al inhibir la replicación del virus o al tener efectos inmunomoduladores, pero es necesario realizar los estudios correspondientes.

Objetivo:

Objetivo principal es evaluar la eficacia de una dosis diaria durante 3 días consecutivos de ivermectina, administrada a pacientes COVID-19 en las primeras 96 horas después del inicio de los síntomas, para reducir la proporción de pacientes con ARN de SARS-CoV-2 detectable por PCR a partir de un hisopado nasofaríngeo en el día 7 después del tratamiento. Se ha escogido el tiempo de 96 horas por ser el periodo en el que se ha documentado la menor replicación viral y por lo tanto la estrategia farmacológica dirigida a la reducción o bloqueo de la transmisión de SARS-CoV-2 podría ser más efectiva. **El objetivo secundario** es evaluar la eficacia de la administración de este régimen para reducir la carga viral de SARS-CoV-2 y mejorar la progresión de los síntomas.

Metodología:

Éste es un ensayo clínico de fase 2a, controlado, aleatorizado, triple ciego con dos grupos paralelos para evaluar la eficacia de ivermectina en reducir la transmisión viral nasal en pacientes con infección por SARS-CoV-2. Se hará una pre-selección con prueba antigénica a pacientes que acudan a los sites con síntomas de menos de 96 horas compatibles con COVID (estimado hasta 1872 pacientes pre-seleccionados). Se reclutarán al ensayo clínico 236 participantes (118 en cada brazo) . Estos pacientes serán aleatorizados para recibir una (1) dosis diaria oral durante 3 días consecutivos de 300 mcg/kg de ivermectina o un placebo equivalente.

2. ANTECEDENTES Y JUSTIFICIÓN DEL ESTUDIO

Ya existen más de 7.2 millones de casos de COVID-19 y más de 410,000 muertes en todo el mundo. A pesar de que se están desarrollando potenciales tratamientos y vacunas para esta enfermedad, se necesitan urgentemente métodos para controlar el COVID-19. Las estrategias basadas en medicamentos se usan habitualmente para controlar enfermedades infecciosas a nivel poblacional (p. ej.; malaria, oncocercosis) [1] y podrían evaluarse para el caso de COVID-19. Se podrían considerar tres estrategias farmacológicas diferentes:

a) **Tratamiento temprano**. Este enfoque requiere identificar pacientes con sospecha de COVID-19 o confirmados tempranamente, a los que se administra un fármaco que reduzca la progresión a enfermedad grave y la muerte por COVID-19. Esta estrategia aliviaría la presión sobre el sistema de salud y disminuiría las muertes indirectas por otras enfermedades que requieran cuidados intensivos.

(b) **Reducción o bloqueo de la transmisión.** Este enfoque implica tratar casos confirmados o sospechosos con un medicamento que reduzca la excreción viral en las vías respiratorias o en las heces. El uso de dicho medicamento a nivel comunitario podría reducir la transmisibilidad y "aplanar la curva" de los casos, incluyendo a aquellos que podrían progresar a enfermedad grave, permitiendo así un mejor funcionamiento del sistema de salud.

(c) **Profilaxis**. Este enfoque consiste en proporcionar un medicamento capaz de prevenir la infección a poblaciones no inmunes vulnerables (p.ej. médicos, personas mayores), siguiendo la misma estrategia que se usa actualmente para proteger a los viajeros de la malaria.

Un medicamento contra el COVID-19 puede que afecte a uno o más de los múltiples mecanismos fisiopatológicos de la enfermedad. SARS-CoV-2, el virus que causa el COVID-19, es un virus ARN positivo de cadena simple similar al SARS-CoV, virus causante de la última epidemia por síndrome respiratorio agudo severo (SARS). El virus SARS-CoV y otros virus tales como el virus de inmunodeficiencia humana (VIH) o el virus de dengue (DENV), han mostrado que su replicación viral depende de la importación nuclear mediada por la importina α/β (2-4). Este mecanismo de transporte celular de proteínas virales hacia el núcleo podría también ser un proceso crucial para la replicación del SARS-CoV-2, y por lo tanto, un potencial blanco farmacológico para detener el COVID-19. Otro mecanismo que podría ser afectado farmacológicamente es la respuesta inmune a la infección. Ya que el virus SARS-CoV-2 es un patógeno nuevo para nuestra especie, las infecciones por este virus están asociadas a un estado hiperinflamatorio e hipercitoquinémico que pueden causar daño pulmonar y falla múltiple de órganos (5). Este perfil inflamatorio está bien descrito e incluye el incremento de interleuquina (IL)-2, IL-7, factor estimulante de colonias de granulocitos (FSC-G), proteína-10 inducible por interferón (PI-10), proteína-1 quimiotractante de monocitos (PQM-1), proteínas inflamatorias de macrófagos y factor de necrosis tumoral (FNT) (6).

La ivermectina es un fármaco antiparasitario ampliamente utilizado para el tratamiento y control de ciertas enfermedades tropicales desatendidas (ETDs) con más de 2.5 billones de dosis distribuidas en los últimos 30 años [8]. Gracias a su excelente perfil de seguridad y potencial actividad más allá de la antiparasitaria, la ivermectina podría considerarse para COVID-19 por dos motivos.

En primer lugar, la ivermectina inhibe la replicación in vitro de varios virus ARN positivos de cadena simple que causan enfermedades en humanos y animales, como el dengue (DENV) [9-11], el Zika [10, 12] y la fiebre amarilla [13, 14], el virus del Nilo Occidental [10], chikungunya [13], encefalitis equina venezolana [15], virus del bosque Semliki [13], virus Sindbis [13], virus del síndrome respiratorio y reproductivo porcino [16] y recientemente SARS-CoV-2 [17]. Esta inhibición se ha observado en un sistema *in vitro* con células Vero a concentraciones micromolares de ivermectina (2.5 uM en SARS-CoV-2 y 17-25 uM en DENV). Mastrangelo et al mostraron que la inhibición de la helicasa NS-3 DENV, una enzima responsable de desenrollar el dsRNA de flavivirus, ocurre a concentraciones de 0.5 uM en DENV [14]. Aún no existen tales datos para SARS-CoV-2. Si bien las concentraciones micromolares no se pueden lograr con dosis aprobadas, la ivermectina podría seguir siendo de utilidad terapéutica si los datos de helicasa NS-3 son alguna indicación. Sorprendentemente, un ensayo clínico en pacientes tailandeses con dengue mostró una reducción en los marcadores moleculares circulantes después de tres administraciones diarias consecutivas de ivermectina 400 ug / kg [18]. Esto no puede explicarse por las concentraciones obtenidas del sistema *in vitro* de células Vero. Si las células Vero ofrecen alguna guía cuantitativa sobre el efecto de la ivermectina, en realidad sería una razón para esperar un efecto más fuerte en el SARS-CoV-2 que en el DENV. Por lo tanto, es concebible que se pueda ver un efecto microbiológico o clínico *in vivo* con una dosis única de 400 mcg/kg (dosis incluida en la ficha de datos de medicamentos de la Unión Europea, UE) dados los niveles triples alcanzados en el tejido pulmonar respecto al plasma una semana después de la administración oral [19, 20], el papel adyuvante de la respuesta inmune y el impacto potencial de los metabolitos de ivermectina activos [20]. Sin embargo, este ensayo tailandés no demostró un efecto clínico, tal vez debido al largo período de incubación de 15 días de la fiebre del dengue, que permitió que gran parte de la replicación viral ocurra antes del inicio de los síntomas y del tratamiento con ivermectina.

En segundo lugar, se ha demostrado que la ivermectina tiene efectos inmunomoduladores al disminuir la producción de TNFa *in vitro* [21, 22] e *in vivo* [21], la producción de IL-1 *in vitro* [21, 22] e *in vivo* [21] y la producción *in vitro* de IL-10 [22]. Además, la ivermectina podría también actuar sobre las células T al aumentar los receptores de superficie *in vivo* [23].

Para nuestro ensayo, proponemos probar el uso de ivermectina, un fármaco antiparasitario seguro con actividad antiviral de amplio espectro y propiedades inmunomoduladoras contra una enfermedad clasificada como una emergencia de salud pública global y para la cual no existe un tratamiento comprobado. Aunque, en el Perú una reciente política de salud en vigencia recomienda el uso de ivermectina como tratamiento específico para COVID-19 bajo criterio médico y previo consentimiento informado. Esta norma fue modificada para delimitar su uso en la dosis previamente aprobada (200 mcg/kg) y sólo para casos COVID-19 leves en pacientes con factores de riesgo para enfermedad grave [24, 25].

Por este motivo, proponemos un ensayo clínico controlado aleatorizado de fase IIa en el Hospital Cayetano Heredia (HNCH) y Hospital Daniel Alcides Carrión que incluirá pacientes con enfermedad COVID-19 temprana. Definimos fase temprana de COVID a aquella comprendida en la primera semana de enfermedad. Los resultados de este ensayo podrían estar disponibles tan pronto como en un mes después de la finalización del reclutamiento. En caso que los resultados fueran positivos y la transmisión viral se reduzca tempranamente, la ivermectina podría usarse a corto plazo para reducir la transmisión a nivel comunitario.

2.1. Producto en Investigación

El producto en investigación (Ivermectina Solución Oral 6 mg/mL) a ser administrado en el estudio Saint-Perú correspondería al producto farmacéutico Noxal, elaborado por el Laboratorio Vitaline Sociedad Anónima Cerrada. Este producto farmacéutico cuenta con RD Nº 2974-2020/Digemid/DPF/UFMNDYO/MINSA y ha sido adquirido para el ensayo en la farmacia de la DIGEMID. Este producto será conservado, etiquetado y dispensado para el ensayo clínico por la farmacia del Hospital Cayetano Heredia.

El régimen de ivermectina propuesto para este estudio es una (1) dosis diaria durante 3 días consecutivos de 300 mcg /kg desde la visita basal. La dosis de ivermectina en este estudio no supera a las dosis actualmente autorizadas por entidades regulatorias internacionales para su comercialización y uso en investigación clínica. La Agencia Europea de Medicamentos (EMA) incluye una dosis única de 400 mcg/kg de ivermectina para el inserto del producto fabricado por Merck (Stromectol®) en los Países Bajos y Francia, y para el producto InfectoPharm (Scabioral®) en Alemania. Además, la Administración Nacional de Medicamentos, Alimentos y Tecnología Médica (ANMAT) aprobó un ensayo clínico que incluye un régimen con una (1) dosis diaría durante 3 días consecutivos de 600 mcg/kg de ivermectina para el tratamiento de trichuriasis en población pediátrica [26].

En este estudio facilitaremos la administración usando la tabla adjunta de dosificación. Dado que la dosis está limitada por la concentración de la solución en gotas de ivermectina (6 mg/ml), los participantes recibirán un número discreto de gotas de acuerdo con su peso en kg como se muestra en la Tabla 1. Cada dosis diaria será de 300 mcg /kg. Consulte el anexo 1 para obtener la tabla completa con la dosis deseada correspondiente al peso corporal.

**Dosis diaria deseada 300 mcg/kg**			
**Peso**	**Dosis total (mg)**	**ml**	**Gotas**
**45**	13.5	2.3	68
**50**	15	2.5	75
**55**	16.5	2.8	83
**60**	18	3	90
**65**	19.5	3.2	98
**70**	21	3.5	105
**75**	22.5	3.8	113
**80**	24	4	120
**85**	25.5	4.3	128
**90**	27	4.5	135
**95**	28.5	4.7	143
**100**	30	5	150
**105**	31.5	5.3	158
**110**	33	5.5	165
**115**	34.5	5.8	173
**120**	36	6	180

Tabla 1. Dosis discretas basadas en tabla de peso.

La eliminación de ivermectina es biliar y a través de procesos metabólicos, presumiblemente por el citocromo P450 3A4 (CYP3A4). En voluntarios sanos, la vida media reportada después de la administración oral varió entre 12 y 36 horas. Esto puede aumentar hasta 54 horas en pacientes con oncocercosis [27]. Además, es concebible que la eliminación se prolongue cuando se inhiben las vías metabólicas relevantes, como con la administración conjunta de inhibidores de CYP3A4 como ritonavir (esto está incorporado dentro de los criterios de exclusión).

El inserto de ivermectina se ha modificado sustancialmente durante 30 años de uso en tratamiento de enfermedades parasitarias y para campañas de salud pública. El régimen de dosificación de ivermectina aprobado por la FDA para el tratamiento comunitario de oncocercosis es de 150-200 mcg/kg (cada 12 meses), aunque la posibilidad de uso trimestral en pacientes individuales también se incluye en el inserto [28]. Para la sarna noruega de moderada a severa, se recomiendan tres dosis de 200 mcg/kg en dos semanas en el inserto australiano [29].

Hay varios estudios publicados del uso de dosis superiores a los 200 mcg/kg para oncocercosis, muchas de ellos alrededor de los 400 mcg/kg y sin efectos colaterales. Ver tabla 2.

Al menos seis ensayos publicados han administrado ivermectina a dosis superiores a 400 mcg/kg. La Tabla 2 resume los datos disponibles de esos ensayos. Estos abarcan 2.662 administraciones independientes de 800 mcg/kg acumulados en una semana y 2.103 administraciones independientes a 800 mcg/kg (dosis única). Solo Kamgno [30] y Smit et al [31] informan los efectos secundarios asociados con el medicamento administrado a estas dosis altas. Los efectos secundarios consistieron en alteraciones visuales leves, transitorias (horas) sin cambios estructurales oculares.

Ref	Dosis única más alta	Freq. Max.	Num. max. de dosis	Dosis total máxima (periodo)	Población	Muestra por dosis	AEs
Awadzi et al 1995 [32]	800 mcg/kg	Única	Única	800 mcg/kg (una vez)	Hombres con infección moderada a severa por Oncocerca	600 mcg/kg x 1: 24 pacientes<br>800 mcg/kg x 1: 17 pacientes	No diferencias con controles que tomaron una dosis de 150 mcg/kg
Awadzi et al 1999 [33]	800 mcg/kg	Días 1 y 4	2	1.600 mcg/kg (4 días)	Hombres adultos infectados con Oncocerca	400-550 mcg/kg x 2: 25 pacientes<br>600-750 mcg/kg x 2: 23 pacientes<br>800-950 mcg/kg x 2: 24 pacientes<br>1600 mcg/kg x 2: 12 pacientes	No diferencias con controles que tomaron una dosis de 150 mcg/kg
Guzzo et al 2002 [34]	2.000 mcg/kg	Días 1, 4 y 7	3	3.273 mcg/kg (una vez por semana)	68 hombres y mujeres sanos, adultos voluntarios	347-594 mcg/kg x 3: 15 voluntarios<br>713-1091 mcg/kg x 3: 12 voluntarios<br>1031-1466 mcg/kg x 1: 12 voluntarios<br>1404-2000 mcg/kg x 1: 12 voluntarios<br>347-541 mcg/kg x 1 (fed): 11 voluntarios	No diferencias con controles
Kamgno et al 2002 [30]	800 mcg/kg	3-monthly	13	8.950 mcg/kg (3 años)	657 hombres adultos infectados con Oncocerca	150 mcg/kg anual: 166 participantes<br>400 then 800 mcg-kg anual: 161 participantes<br>150 mcg/kg 3-mensual: 161 participantes<br>400 then 800 mcg-kg 3-mensual: 158 participantes	The high dose group reported a statistically higher rate of transitory mild and subjective visual side effects.<br>(No structural explanation).<br>For all other AE comparable rates reported in all groups.
Munoz et al 2018 [35]	705 mcg/kg (dosis única de 36 mg)	Único	Única	705 mcg/kg (una vez)	54 hombres y mujeres sanos, adultos voluntarios	276-352 mcg/kg x 1: 18 voluntarios<br>227-272 mcg/kg x 1: 19 voluntarios<br>157-225 mcg/kg x 1: 20 voluntarios<br>553-705 mcg/kg x 1: 18 voluntarios<br>455-545 mcg/kg x 1: 19 voluntarios<br>315-450 mcg/kg x 1: 20 voluntarios	No diferencias significativas comparadas con una dosis de 200 mcg/kg ajustada al peso
Smit et al 2018 [31]	600 mcg/kg	Días 1, 2 y 3	3	1.800 mcg/kg (3 días)	141 hombres y mujeres adultos con malaria no complicada	300 mcg/kg x 3: 48 participantes<br>600 mcg/kg x 3: 47 participantes	No diferencias en EAS. El régimen con 3 dosis de 600mcg/kg de ivermectina tuvo 15% más AE reflejando predominantemente un deterioro visual transitorio. Un caso de anafilaxis fue reportado en este estudio

Tabla 2. Estudios de ivermectina que evalúan la seguridad de dosis superiores a 400 mcg / kg

Reacciones adversas

Cuando se usa ivermectina en pacientes con estrongiloidiasis (dosis de 200 mcg / kg), se han descrito los siguientes efectos secundarios clasificados por sistema corporal [28]:

- Cuerpo en general: astenia/fatiga (0.9%), dolor abdominal (0.9%)
- Gastrointestinales: anorexia (0.9%), estreñimiento (0.9%), diarrea (1.8%), náuseas (1.8%), vómitos (0.9%)
- Sistema nervioso/psiquiátrico: mareos (2.8%), somnolencia (0.9%), vértigo (0.9%), temblor (0.9%)
- Piel: prurito (2.8%), erupción cutánea (0.9%) y urticaria (0.9%).

Cuando se usa ivermectina **en pacientes con oncocercosis** (dosis 150-200 mcg/kg) se han descrito los siguientes efectos secundarios.

- Reacciones de Mazzoti durante los primeros 4 días después del tratamiento, que incluyen artralgia / sinovitis (9.3%), agrandamiento o sensibilidad de los ganglios linfáticos (hasta 13.9%), prurito (27.5%), erupción urticaria (22.7%) y fiebre (22.6)
- Empeoramiento de las condiciones oftalmológicas preexistentes: limbitis (5.5%), opacidad puntiforme (1.8%).
- Además, se reportaron las siguientes reacciones adversas clínicas como posible, probable o definitivamente relacionadas con el medicamento en ≥1% de los pacientes: edema facial (1.2%), edema periférico (3.2%), hipotensión ortostática (1.1%) y taquicardia (3.5%). Dolor de cabeza y mialgia relacionados con las drogas ocurrieron en <1% de los pacientes (0.2% y 0.4%, respectivamente).

También vale la pena recalcar que estos efectos secundarios se han asociado con la lisis parasitaria en oncocercosis, más que con la ivermectina en sí.

En pacientes que reciben dosis acumulativas superiores a 800 mcg/kg en una semana, se han descrito trastornos inespecíficos leves (que no impiden las actividades diarias), transitorios (que duran menos de 24 horas), visuales (visión borrosa, visión de túnel).

Cuando se usa ivermectina en pacientes con Loa concomitante con alta carga parasitaria (> 15,000 mf / ml), se han descrito los siguientes efectos secundarios en relación con la lisis rápida del parásito:

Clínicos: encefalopatía en 0.01-0.11% de la población tratada, y un síndrome que incluye confusión, letargo y coma [36].

Hallazgos de pruebas de laboratorio informados como posible, probable o definitivamente asociados con ivermectina:

- Elevación de las enzimas hepáticas (ALT y/o AST) (2%)
- Disminución del recuento de leucocitos (3%).
- Leucopenia y anemia (<1%).

También se han reportado las siguientes reacciones adversas a la ivermectina desde la autorización de su comercialización (para usos diferentes a SARS-CoV 2) de la FDA:

- Hipotensión (principalmente hipotensión ortostática)
- Empeoramiento del asma bronquial.

- Necrolisis epidérmica tóxica
- Síndrome de Stevens-Johnson
- Convulsiones
- Hepatitis
- Elevación de las enzimas hepáticas.
- Elevación de bilirrubina

Embarazo

A dosis altas, que van desde 10 a 80 veces las dosis y regímenes terapéuticos humanos, la ivermectina es un teratógeno conocido en mamíferos y está clasificada por la FDA como un medicamento de categoría C. Durante las campañas comunitarias para oncocercosis con dosis más bajas dentro del inserto de la Agencia de Medicinas Europea (EMA), se excluyen las mujeres embarazadas visualmente, pero no se realizan pruebas formales de forma rutinaria en mujeres en edad fértil. Hay varios informes de tratamiento inadvertido durante el embarazo. Un reciente metanálisis de Nicolas et al [37] analizó datos de 893 embarazos expuestos inadvertidamente (97 en el primer trimestre) y concluyó que no hay pruebas suficientes para comprender la seguridad de la ivermectina durante el embarazo.

Es por ello que embarazo es un criterio de exclusión para este estudio.

Justificación de dosis de ivermectina

El estudio se propone como un ensayo clínico aleatorizado de fase IIa. La ivermectina ha demostrado actividad antiviral e inmunomoduladora *in vitro,* aunque aún existe incertidumbre acerca de si el efecto inmunomodulador podría aumentar los síntomas de COVID-19. Las dosis comúnmente usadas como antiparasitario son de alrededor de 200 mcg/kg, sin embargo, la dosis activa antiviral *in vitro* que se ha documentado es casi 50 veces esta dosis. Los estudios han mostrado que a dosis de 800 mcg/kg (acumulada) se evidencian toxicidad transitoria ocular. El estudio ha considerado usar una (1) dosis diaria durante 3 días consecutivos de ivermectina para así maximizar la seguridad de los participantes y maximizar la dosis en un rango seguro. El esquema de una (1) dosis diaria durante 3 días consecutivos de 300 mcg/kg dará como resultado una excreción completa en 5-7 días, lo cual disminuye también los riesgos relacionados con la inmunomodulación. Se incluirán como pacientes sólo aquellos con enfermedad leve y temprana.

Al tratar a los pacientes dentro de las 96 horas posteriores a los síntomas, nuestro objetivo es reducir la replicación viral temprana y mejorar la potencial eficacia de la ivermectina. La variable principal de valoración es microbiológica (proporción que negativizan PCR en 7 días), lo que hace que la evaluación sea objetiva. Los objetivos secundarios incluyen variables indirectas, tales como la carga viral y la evaluación de la respuesta inmune, que reducirán el riesgo de error tipo II y garantizarán la evaluación adecuada de un medicamento con posibles beneficios.

Placebo

El placebo será visiblemente idéntico. Ni la persona que administra el medicamento/placebo ni el paciente podrán diferenciar el producto en investigación, de modo que se mantiene el ciego. Es aceptable usar placebo como control en este ensayo, ya que actualmente no existe un medicamento aprobado para el

tratamiento del SARS-CoV-2. La formulación del placebo es exactamente la misma que la de la solución de ivermectina excepto por la ausencia del principio activo Ivermectina.
El placebo será preparado por la co-investigadora, farmacéutica del estudio, Dra. Patricia León (PL). El procedimiento se realizará en la farmacia del Hospital Nacional Cayetano Heredia, que tiene el servicio de farmacotecnia, reconocido por la DIGEMID y es dirigido por la Dra. León, que es además la jefa del departamento.

Otros fármacos permitidos durante el ensayo
Se permiten medidas de apoyo al paciente, como el uso de antipiréticos e hidratación, siempre que no interfieran con los criterios de exclusión. Los participantes pueden continuar tomando cualquier fármaco y terapia no farmacológica que no esté en la lista de los excluidos para el ensayo.

2.2. Enfermedad bajo estudio

COVID-19 es la enfermedad causada por la infección por SARS-CoV-2 y actualmente está clasificada como una pandemia. Se sabe que COVID-19 puede causar un estado hiperinflamatorio severo asociado con neumonía, insuficiencia respiratoria y multiorgánica, particularmente en pacientes mayores de 60 años y con comorbilidades. Actualmente no existe un tratamiento específico para COVID-19.

La población del estudio serán pacientes adultos con no más de 96 horas de síntomas típicos de COVID-19 que acuden al HNCH (ver criterios de exclusión).

El grupo de control recibirá placebo. No existen datos actuales sobre la eficacia de ivermectina contra el virus *in vivo*, por lo que el uso de placebo en el grupo de control en nuestro estudio está éticamente justificado. Los participantes en el ensayo tendrán enfermedad leve y serán monitoreados cercanamente durante 21 días, y especialmente durante la primera semana, incluso serán evaluados por vía telefónica diariamente para la toma de parámetros clínicos. En caso de que se evidencie signos alerta o empeoramiento clínico, el paciente recibirá las indicaciones para ir al HNCH para el manejo correspondiente.

2.3. Evaluación de la relación beneficio/riesgo para los participantes en el ensayo clínico

La ivermectina tiene un perfil de seguridad bien documentado para la dosis propuesta y su posible actividad inmunomoduladora y antiviral contra el SARS-CoV-2 supera el riesgo de eventos adversos relacionados con el medicamento. Sin embargo, es posible que los participantes no obtengan un beneficio directo por tomar ivermectina.

3. OBJETIVOS DEL ESTUDIO

3.1 Objetivo principal

Determinar la eficacia de una (1) dosis diaria durante 3 días consecutivos de ivermectina, administrada a pacientes COVID-19 en las primeras 96 horas después del inicio de los síntomas, para reducir la proporción de pacientes con ARN de SARS-CoV-2 detectable por PCR a partir de un hisopado nasofaríngeo en el día 7 después del tratamiento. Se ha escogido el tiempo de 96 horas por ser el periodo en el que se ha documentado la menor replicación viral y por lo tanto la estrategia farmacológica dirigida a la reducción o bloqueo de la transmisión de SARS-CoV-2 podría ser más efectiva.

Variable principal de valoración

Proporción de pacientes con un PCR positivo de SARS-CoV-2 de un hisopado nasofaríngeo el día 7 después del tratamiento

3.2 Objetivos secundarios

1. Evaluar la eficacia de la ivermectina para reducir la carga viral de SARS-CoV-2 en el hisopado nasofaríngeo en los días 4, 7, 14 y 21 después del tratamiento.
2. Evaluar la eficacia de la ivermectina en la mejora los síntomas en pacientes tratados.
3. Evaluar la proporción de seroconversiones el día 21 en pacientes tratados.
4. Evaluar la seguridad de la ivermectina a la dosis propuesta.
5. Determinar la magnitud de la respuesta inmune contra el SARS-CoV-2.
6. Evaluar la presencia de helmintos en visita basal y en visita día 14.

Variables secundarias de valoración

1. Carga viral media según lo determinado por el umbral del ciclo de PCR (Ct) al inicio y en los días 4, 7, 14 y 21.
2. Proporción de pacientes con fiebre y tos en los días 4, 7, 14 y 21, así como la proporción de pacientes que progresan a enfermedad grave o muerte durante el ensayo.
3. Proporción de pacientes con seroconversión en el 7, 14 y día 21.
4. Proporción de eventos adversos reportados relacionados con las drogas.
5. Niveles de IgG, IgM, IgA medidos por Luminex y otras medidas inmunológicas que se realizarán posteriormente en plasma guardado, cuando se consiga financiamiento adicional
6. Proporción y carga parasitaria de helmintos intestinales mediante método de sedimentación espontánea en tubo descrita por Tello en visita basal y día 14.

4. DISEÑO DE ENSAYO

Este es un ensayo controlado aleatorizado triple ciego ivermectina-placebo para determinar la eficacia de una (1) dosis diaria durante 3 días consecutivos de ivermectina, administrada a pacientes COVID-19 en las primeras 96 horas después del inicio de los síntomas, para reducir la proporción de pacientes con ARN de SARS-CoV-2 detectable por PCR a partir de un hisopado nasofaríngeo en el día 7 después del

tratamiento.. Estimamos que, al negativizar las secreciones de las vías respiratorias en los casos primarios durante la etapa precoz de la enfermedad, se podría reducir la transmisión y la cantidad de casos secundarios subsecuentes en la comunidad. El sustento para esto es que múltiples series de pacientes con COVID-19, tanto severa como no-severa e incluyendo asintomáticos muestran una positividad del 100% al día 7 posterior al diagnóstico [38-42]. Un 44% (25-76%) de los casos secundarios de COVID-19 son infectados durante la etapa pre-sintomática o sintomática precoz [10, 39]. Recientemente, Chacour ha realizado un ensayo piloto, aleatorizado, doble ciego y controlado con placebo para evaluar la eficacia de una dosis única de ivermectina para reducir la transmisión del SARS-CoV-2 cuando se administra poco después del inicio de la enfermedad. Este artículo reporta una positividad de 92% en RT-PCR para el gen E a una semana de tratamiento en pacientes tratados con ivermectina, versus 100% en el grupo placebo (RR: 0.92, IC 95% 0.77 – 1.09), esta diferencia encontrada de aproximadamente 10% no fue significativa entre grupos. También reporta que el grupo de ivermectina tuvo cargas virales más bajas pero que las diferencias no fueron estadísticamente significativas y que estos pacientes del grupo de ivermectina se recuperaron antes de la hiposmia / anosmia (76 frente a 158 pacientes-día; p <0,001). Una recomendación importante del estudio es continuar la investigación con un mayor número de pacientes, dado que su estudio incluyó 24 pacientes totales, 12 en cada grupo [44]. Hemos considerado un 10% de reducción al día siete basados en el estudio de Chaccour. El ensayo se realizará en el Hospital Nacional Cayetano Heredia y Hospital Daniel Alcides Carrión.

4.1. Diseño

Los participantes serán aleatorizados para recibir una (1) dosis diaria durante 3 días consecutivos de 300 mcg/kg de ivermectina o placebo. El epidemiólogo (CC) generará una lista de números correlativos con asignación aleatoria a dos grupos de tratamiento (a y b), sin saber qué tratamiento será asignado a cada letra. La asignación se hará por bloques completos aleatorizados de tamaño 4. Esta lista será entregada directamente a la farmacéutica. De manera independiente la investigadora principal (PG) asignará de manera aleatoria la intervención (ivermectina) a uno de los dos grupos (a o b) lanzando una moneda, sin tener acceso al listado de aleatorización, e informará el resultado de este proceso a la farmacéutica. La farmacéutica (PL) preparará los frascos de tratamiento rotulados de acuerdo con el listado de aleatorización preparado por el epidemiólogo y según la asignación de tratamientos entregado por la investigadora principal.

Los potenciales participantes, adultos de 18 años o más, con no más de 96 horas de síntomas (fiebre, tos, anosmia etc.) y que no hayan tomado ivermectina antes de acudir al hospital, serán invitados a una primera fase de preselección, en la cual se les explicará el estudio, firmarán un consentimiento de preselección (Anexo 2) y se les ofrecerá la toma de la prueba antigénica nasofaríngea, la prueba rápida serológica, la toma de prueba de embarazo en mujeres de edad reproductiva, evaluación de la saturación de oxígeno y auscultación en busca de crepitantes. Los resultados de las pruebas antigénicas toman 15 minutos. Si el participante es positivo y cumple con los otros criterios de inclusión y no tiene criterios de exclusión, se le invitará a participar en el ensayo clínico. El investigador del estudio o un médico asignado explicará los detalles del estudio, se le invita a participar y se le ofrece el consentimiento. Se tomará una foto del consentimiento informado de preselección y consentimiento del ensayo clínico para los récords del investigador y el paciente se quedará con el/los documentos originales. Los pacientes reclutados pasarán a la visita basal. Los datos obtenidos de la visita basal se llenarán en la **Ficha de Visita Basal** (anexo 3). Cabe resaltar que en todas las fichas del estudio se usará un Código de Investigación del Participante (CIP) en lugar de los nombres del participante, para velar por la confidencialidad de sus datos. Adicionalmente durante esta visita se obtendrá información sobre la medicación actual y pasada de hasta una semana y se registrará en la **"Ficha de Medicación Concomitante"** (anexo 4).

Inmediatamente después se tomará la prueba de PCR. A continuación, el investigador o médico asignado recepcionará el frasco de "tratamiento" previamente aleatorizada en la farmacia y administrará la primera dosis al participante dentro de las instalaciones del centro de investigación mediante el tratamiento directamente observado (DOT) y le entregará el frasco. Se registrará en la Ficha Basal la hora exacta de administración, y se supervisará la tolerancia inmediata por 30 minutos. Si se presentase algún evento adverso, éste será registrado en la **"Ficha de Eventos Adversos"** (anexo 5)

En el **"Día 2"** y **"Día 3"** el participante deberá tomar la dosis diaria correspondiente y se le llamará por teléfono para confirmar que ha tomado la dosis correspondiente y guarde los frascos vacíos para mostrarlos en la visita 4. El médico del estudio registrará la hora de la toma de los días 2 y 3 en la "**Ficha de visita basal"**. Se monitorearan también presencia de eventos adverso luego de la toma, y estos serán registrado en la **"Ficha de Eventos Adversos".**

	**Dia 1**<br><br>**Preselec ción**	**Día 1**<br><br>**Recluta miento**	**Día 2**	**Día 3**	**Día 4**<br><br>**Visita 2**	**Día 7**<br><br>**Visita 3**	**Día 14**<br><br>**Visita 4**	**Día 21**<br><br>**Visita 5**
Consentimiento informado para preselección	X							
Preselección<br>- Prueba antigénica (hisopado nasofaríngeo)<br>-Saturación de oxígeno<br>-Crepitantes pulmonares<br>-Prueba rápida serológica<br>-Prueba de embarazo en orina<br>-Otros criterios según anexo 3	X							
Consentimiento informado para el ensayo clínico		X						
Consentimiento informado de almacenamiento de muestras para uso futuro		X						
Ficha de Visita Basal		X						
Ficha de Medicación concomitante		X						
Heces para estudio de helmintos		X					X	
Hisopado nasofaríngeo (PCR para SARS-CoV-2)		X			X	X	X	X
Toma de muestra de sangre para inmunología (para laboratorio de Hospital)		X				X	X	X
Prueba rápida serológica SARS-CoV-2						X	X	X
Aleatorización, primera dosis y entrega de medicamento		X						
Ficha de Eventos Adversos		X<br>(30 min post-dosis)	X--- X					
Ficha de evaluación clínica diaria*		X--- X						
Segunda dosis			X					

Tercera dosis				X				
Ficha visita de seguimiento					X	X	X	X
	*Se realizará remotamente todos los días desde el día 1 al 21							

Figura 1. Cronograma de actividades de estudio.

Los participantes permanecerán en el ensayo por un período de 21 días. En interés de la salud pública y de contener la transmisión de la infección, las visitas de estudio del **"Día 4", "Día 7", "Día 14"** y **"Día 21"** se llevarán a cabo en el domicilio del participante o donde se encuentre el paciente (ejm. en el caso que esté hospitalizado) por un médico. Estas visitas serán realizadas cumpliendo todos los estándares de bioseguridad recomendados para la contingencia COVID-19, por medio de uso de EPP. En estas visitas el médico tomará los parámetros clínicos y completará un cuestionario de síntomas COVID-19, y otra medicación concomitante (si hubiera) y se tomarán las muestras biológicas correspondientes con el cronograma de visitas del estudio. Los datos colectados serán registrados en la **"Ficha visita de seguimiento"** (anexo 6) y **"Ficha de Medicación Concomitante"** (anexo 4).

Adicional a las visitas domiciliarias (o donde se encuentre el paciente), el estudio incluye la colección de datos clínicos con una frecuencia diaria. El coordinador del estudio o el médico designado realizará diariamente la evaluación de síntomas y signos por vía telefónica guiada por la **"Ficha de evaluación clínica diaria"** (anexo 7). Si durante la participación apareciese algún EA, se registrará su denominación, grado de severidad, fecha de inicio, duración y medidas de mitigación usadas en la **"Ficha de Eventos Adversos"** (anexos 5).

Dado que el laboratorio para este estudio procesa las muestras en un tiempo máximo de 24-48 horas, hemos previsto el escenario que algunos paciente puedan tener un PCR basal negativo después de haber tomado recibido la intervención o placebo (al menos una dosis), aunque esta posibilidad se reduce de manera significativa al usar la prueba de antígeno . En ese caso comunicaremos con el participante; le haremos saber sobre sus resultados, le indicaremos que pare la medicación y la descarte; y le informaremos que allí termina su participación en el estudio, como se lo habíamos explicado durante el proceso de consentimiento informado, y le ofreceremos enviarle los resultados virtualmente.

Se continuará la aleatorización y reclutamiento hasta alcanzar la meta de reclutamiento de pacientes COVID-19 PCR positivos.

Con respecto al producto en investigación, se ha considerado una cantidad adicional de ivermectina y placebo para el estudio.

Por otro lado, a la fecha, el Ministerio de Salud (MINSA) recomienda la indicación médica de ivermectina, incluso en su formulación magistral, para el tratamiento sindrómico de COVID-19 leve durante la etapa precoz de la enfermedad. Por lo que, los pacientes con PCR negativo que sean retirados anticipadamente, no habrán recibido un tratamiento distinto al actualmente recomendado a nivel nacional.

Con respecto al seguimiento de la seguridad, se dará seguimiento los Eventos Adversos Serios (EAS) relacionados con ivermectina hasta que se resuelvan o hasta 30 días despúes de la visita final del participante, lo que ocurra primero. Además, se hará seguimiento a todos los otros EAS considerados como no-relacionados a ivermectina hasta la visita final del participante o durante un período específico a discreción del investigador. El estudio finalizará cuando el último paciente aleatorizado haya completado el estudio, se hayan realizado todas las visitas planificadas y se hayan resuelto cualquier inconsistencia de datos.

Un paciente puede interrumpir su participación en el estudio en cualquier momento y por cualquier motivo. El investigador principal también puede retirar a un paciente del estudio si considera que es lo mejor para el paciente.

5. PRE-SELECCIÓN DE LOS PACIENTES Y SELECCIÓN DE LOS PACIENTES PARA EL ENSAYO

5.1. Criterios para invitar a participar en la pre-selección

1. Pacientes que acuden con síntomas COVID-19 típicos (fiebre, tos, anosmia, y otros etc.) de no más de 96 horas de evolución.
2. De 18 años de edad a más.
3. No haber tomado ivermectina antes de asistir al hospital
4. No tiene historia conocida de alergia a la ivermectina
5. El paciente es capaz de dar consentimiento para participar en el estudio.
6. No usa fármacos inhibidores de CYP 3A4 o P-gp, tales como quinidina, amiodarona, diltiazem, espironolactona, verapamilo, claritromicina, eritromicina, itraconazol, ketoconazol, ciclosporina, tacrolimus, indinavir, ritonavir o cobicistat o fármacos sustratos críticos de CYP3A4, como warfarina.

Estos pacientes serán invitados a participar en la pre-selección, se les explicará el estudio y si aceptan firmarán el consentimiento informado de pre-selección.

5.2. Criterios de exclusión

1. Neumonía por COVID-19
    - Diagnosticado por el médico (saturación < 95% o crepitantes)
2. Prueba de embarazo positiva para mujeres en edad fértil
3. IgG positiva contra SARS-CoV-2 por prueba rápida
4. Prueba de antígeno negativa

5.3. Criterios para la retirada del estudio

Se informará a los pacientes que pueden retirarse del estudio en cualquier momento sin dar ninguna explicación. La retirada también puede tener lugar por cualquiera de los siguientes motivos:

- El PCR basal sea negativo
- Decisión tomada por el paciente.
- El paciente no puede completar las visitas del estudio debido a circunstancias imprevistas.
- Desarrollo de otros trastornos que, en opinión del investigador y en el mejor interés del paciente, hacen aconsejable que abandone el estudio.
- Administración de un tratamiento concomitante no permitido (ver 6 criterios de exclusión).

- El investigador cree que el paciente puede beneficiarse de un tratamiento diferente.
- Pérdida de seguimiento.

El investigador intentará garantizar que el paciente acepte la visita final del ensayo. Los datos de los pacientes retirados se recopilarán hasta esta visita final. Si la retirada es el resultado de un evento adverso grave relacionado con ivermectina o SARS-CoV-2, los datos se recopilarán hasta que el paciente se recupere o se estabilice. La retirada del estudio se documentará en la historia clínica del paciente, indicando los motivos de la decisión. Los participantes serán reemplazados si la retirada es por una razón diferente a la falta de eficacia del tratamiento o la aparición de eventos adversos. Se harán todos los esfuerzos para mantener el número total de pacientes evaluables según el protocolo. Para ello se hará un seguimiento del paciente por teléfono, llenando también el cuestionario diario de síntomas, se harán las visitas correspondientes según el protocolo, y se conversará con los pacientes para anticipar si durante el periodo del estudio decidieran cambiar de domicilio o viajar para asegurarnos continuar en contacto.

6. DESCRIPCIÓN DEL TRATAMIENTO DEL ENSAYO

6.1. Método de asignación de tratamiento

Éste es un ensayo clínico triple ciego, aleatorizado y de centro único con dos grupos paralelos que evalúa la eficacia de la ivermectina en la negativización del PCR en pacientes en fase temprana de COVID-19. Los pacientes elegibles serán inscritos en el estudio bajo la supervisión del investigador o personal designado. Serán aleatorizados para recibir una (1) dosis diaria durante 3 consecutivos de 300 mcg/kg de ivermectina o un placebo equivalente. La persona que administra el medicamento observará que el participante tome la primera dosis diaria bajo la modalidad de "tratamiento directamente observado" (DOT). El placebo coincidirá con la ivermectina en apariencia. El equipo clínico permanecerá cegado al tratamiento recibido por todos los participantes. El equipo que analizará la data también será ciego a la asignación del tratamiento vs placebo.

6.2 Información farmacéutica

El investigador se responsabilizará y tomará todas las medidas para mantener los registros apropiados y garantizar el suministro, manejo, almacenamiento, distribución y uso apropiados de estos materiales de acuerdo con el protocolo y el Reglamento de Ensayos Clínicos del Perú. Consulte la Tabla 3 para obtener información farmacéutica referente a ivermectina.

	Ivermectina
Formulación de dosis	Ivermectina 6mg/ml<br>Solución oral en gotas
Fuerza de dosis unitaria (s)	300 mcg/kg

Vía de administración	Ivermectina oral en una dosis diaria durante 3 días consecutivos
Almacenamiento	Almacenar a una temperatura no mayor de 30 C. Proteger de la luz.
Envasado y etiquetado	Envase en frasco con rotulación respectiva con el nombre del estudio y número de asignación correlativa.
Instrucciones de dosificación[(a)]:	El PI se administrará en una (1) dosis diaria durante 3 días consecutivos de 300 mcg/kg siguiendo la dosificación descrita en la tabla 1.
Lista de excipientes	Propilenglicol<br>Aspartamo<br>Metilparabeno<br>Propilparabeno<br>Sorbitol 70%<br>Esencia de uva
Incompatibilidades	Muy raramente, reportes post-comercialización han informado un incremento del INR (International Normalised Ratio) cuando se administró conjuntamente ivermectina con warfarina. Consulte los criterios de exclusión y la tabla 6.

Table 3. Información farmacéutica de la ivermectina

La farmacéutica rotulará los frascos del producto y preparará el placebo en la farmacia del Hospital Nacional Cayetano Heredia, en una estación de trabajo en condiciones asépticas y libre de elementos que no estén relacionados, según las normas locales. Las medidas de seguridad para la preparación y el manejo incluyen ropa protectora, guantes y el uso de gavetas de seguridad. La farmacéutica (PL) preparará los frascos de tratamiento rotulados de acuerdo con el listado de aleatorización preparado por el epidemiólogo y según la asignación de tratamientos entregado por la investigadora principal.

6.3. Identidad de producto

La ivermectina es un agente antihelmíntico semisintético derivado de la bacteria del suelo Streptomyces avermitilis. La ivermectina es la mezcla de 22,23-dihidro-avermectina B1a (al menos 90%) y 22,23-dihidro-avermectina B1b (menos del 10%); forma parte de las avermectinas, que son agentes antiparasitarios de amplio espectro que tienen un modo de acción único. Los compuestos de esta clase se unen selectivamente y con alta afinidad a los canales de iones de cloruro activados por glutamato que se producen en las células nerviosas y musculares de invertebrados. Esto conduce a un aumento en la permeabilidad de la membrana celular a los iones de cloruro con hiperpolarización de las células nerviosas o musculares, lo que resulta en parálisis y muerte del parásito. Los compuestos de esta clase también pueden interactuar con otros canales de cloruro activados por ligandos, como los activados por el neurotransmisor ácido gamma-aminobutírico (GABA).

La actividad selectiva de los compuestos de esta clase es atribuible a que algunos mamíferos no tienen canales de cloruro dependientes de glutamato y que las avermectinas tienen una baja afinidad por los canales de cloruro dependientes de GABA de mamíferos. Además, la ivermectina no cruza fácilmente la barrera hematoencefálica intacta en los humanos.

6.4. Riesgos potenciales

Consulte la sección 2.1.

6.5. Administración

El PI se administrará en el área designada para pacientes con COVID-19 en el "Día 1", y en el domicilio del paciente en el "Día 2" y "Día 3".

6.6. Empaque y rotulado

El producto en investigación será suministrado por el la Farmacia del HNCH, empaquetados y rotulados de acuerdo con los requisitos estándar para ensayos clínicos. La cantidad total de frascos del PI para una dosis diaria durante 3 días consecutivos se preparará de acuerdo con el peso de cada participante (tabla 1). El farmacéutico rotulará la ivermectina y el placebo, y ambos estarán en frascos opacos indistinguibles. El medicamento del estudio se rotulará de acuerdo con las recomendaciones del Reglamento de Ensayos Clínicos del Perú.

6.7. Manejo y almacenamiento

La ivermectina y el placebo serán recepcionados por el investigador y serán guardados en el site. Se registrará la fecha y hora de entrada del producto de investigación, así como la información del lote y fabricante. Hasta que se distribuya a los participantes, el producto en investigación se almacenará en un área cerrada con seguridad, accesible solo para personal autorizado.

6.8. Dispensación

Solo los participantes inscritos en el estudio pueden recibir la intervención del estudio y solo el personal autorizado del centro de investigación puede suministrar o administrar la intervención del estudio. El investigador principal acepta que el medicamento del estudio será dispensado por el investigador o médico de estudio asignado en el Acuerdo de Investigador o por sus representantes calificados. El investigador, los sub-investigadores o los representantes calificados también acuerdan que los medicamentos del estudio se dispensarán sólo a los sujetos del estudio que hayan dado su consentimiento informado y hayan cumplido con todos los criterios de elegibilidad y de acuerdo con las instrucciones proporcionadas.

La asignación de tratamiento para ivermectina/placebo tomará lugar dentro del centro de investigación en el "Día 1" y se le entregará al paciente para que tome las dosis diarias de los días 2 y 3. La administración de las tres tomas del PI se registrará utilizando la "Ficha de Visita Basal".

6.9. Responsabilidad

El investigador o la persona designada es responsable de mantener exactos los registros de dispensación del tratamiento del estudio durante todo el estudio clínico. El registro de responsabilidad del tratamiento del estudio incluye información del producto en investigación, el número de viales asignados, el PI

utilizado, el lote y la fecha de vencimiento de dichas soluciones, la dosis, la identificación del paciente, el cantidad que contiene el frasco dispensado, la persona responsable del registro de responsabilidad.

Los registros electrónicos de responsabilidad del centro de investigación estarán disponibles para monitorear las visitas. Todos los registros de dispensación y rendición de cuentas deben almacenarse de acuerdo con las regulaciones de la institución patrocinadora.

El registro de responsabilidad de los productos recepcionados y despachados, o destruidos será mantenido con exactitud por el centro de investigación.

6.10. Cumplimiento de intervención de estudio

Los participantes recibirán la intervención del estudio directamente por un médico designado en el estudio. La fecha y la hora de cada dosis diaria administrada se registrarán en la “Ficha de Visita Basal”.

La solución de ivermectina o placebo se tomarán por vía oral en el centro de investigación, y posteriormente en el domicilio del participante.

6.11. Eliminación y destrucción

Cualquier medicamento no utilizado o material de desecho debe eliminarse de acuerdo con el reglamento de ensayos clínicos. Las aprobaciones para la destrucción de fármacos en el centro de investigación se archivarán en la carpeta de la farmacia además del Certificado de Destrucción de acuerdo con la política de destrucción vigente.

6.12. Medicación / dispositivos concomitantes

Cualquier medicamento concomitante importante que el participante esté recibiendo al momento de la inscripción o que reciba durante el estudio debe registrarse en el Ficha de medicación concomitante, junto con:

- Motivo de uso
- Fechas de administración, incluidas las fechas de inicio y finalización.
- Información sobre la dosis y la frecuencia

Si hay alguna pregunta con respecto a la medicación concomitante, se podrá contactar al médico personal del participante.

Fármacos o tratamientos permitidos (incluidos los medicamentos de rescate) y no permitidos antes y / o durante el ensayo.

Los participantes pueden continuar tomando cualquier medicamento y terapia no farmacológica que no esté en la lista de medicamentos excluidos para el ensayo.

Los fármacos inhibidores de CYP3A o P-gp u otros medicamentos que pueden interferir con el producto en investigación se encuentran dentro de los criterios de exclusión. Consulte la tabla 4 para más detalles.

Clase	Fármaco	Fundamento
Antiarrítmico / Antihipertensivo	Quinidina	Puede aumentar la exposición a la ivermectina al inhibir su metabolismo y excreción o al competir con los CYP o la P-gp.
	Amiodarona	
	Diltiazem	
	Espironolactona	
	Verapamil	
Antibióticos-Macrólidos	Claritromicina	
	Eritromicina	
Agentes antifúngicos	Itraconazol	
	Ketoconazol	
Inmunosupresores	Ciclosporina	
	Tacrolimus	
Terapia anti HIV	Indinavir	
	Ritonavir	
	Cobicistat	
Anticoagulante	Warfarina	
Esteroides	Dexametasona	

Tabla 4 Tratamiento concomitante utilizado como criterio de exclusión

6.13. Escala de dosis

Se administrará una (1) dosis diaria durante 3 días consecutivos, éstas serán equivalentes a 300 mcg/kg.

6.14. Modificación de dosis y retraso

No habrá modificación en las dosis.

6.15. Toxicidad limitante de dosis

No se superará dosis aprobada por EMA o ANMAT.

6.16. Sobredosis

En caso de una sobredosis, el paciente será monitoreado cercanamente, tratado sintomáticamente y se tomarán las medidas de apoyo según sea necesario.

6.17. Interrupción del tratamiento

Se interrumpirá el tratamiento en caso de aparición de un EAS o SUSAR o alguna razón clínicamente relevante para el investigador.

7. EVALUACIÓN DE RESPUESTA

7.1. Plan del estudio y procedimientos

7.1.1. Obtención del consentimiento informado de preselección, del ensayo clínico y almacenamiento de muestras

Parte del proceso del consentimiento informado consiste en explicar claramente los propósitos, métodos, objetivos y riesgos del estudio al paciente. Una vez se expliquen los detalles del estudio y el proceso de preselección en su totalidad al paciente, se procederá a que el participante e investigador firmen el formulario de consentimiento informado de pre-selección y registren sus iniciales y fechas (anexo 1). Si el participante es seleccionado, se procederá a que el participante e investigador firmen el consentimiento informado del estudio. En un consentimiento informado adicional, preguntaremos al participante si desea autorizar el almacenamiento de sus muestras de sangre obtenidas en las visitas basal, 7, 14 y 21 para uso futuro (anexo 9). Estas muestras se almacenarán en el Laboratorio de Enfermedades Tropicales del Hospital Nacional Cayetano Heredia, el mismo laboratorio donde se procesan todos los exámenes de laboratorio de este estudio. La elección será tomada y registrada por el participante sin alguna influencia del personal de estudio.

Durante este período de pandemia de SARS-CoV-2, se considera importante contar con "mecanismos alternativos apropiados para garantizar que el estudio se lleve a cabo evitando el riesgo de contagio y permitiendo dejar registro de la voluntad del participante. Por este motivo, en este estudio no se archivarán los formularios firmados de consentimiento informado dentro del site. En su lugar se tomará una fotografía de los consentimientos firmados para los récords del investigador, y se le entregarán los originales al participante. El investigador debe documentar por escrito en la historia clínica del paciente que el consentimiento fue recibido.

Solo después de completado el proceso de consentimiento informado se procederá a hacer efectiva la participación y proseguirá con las actividades y procedimientos del estudio, incluyendo el completar la evaluación de elegibilidad. El método utilizado para obtener y documentar el consentimiento informado y los contenidos del mismo cumplen con las indicaciones del Reglamento de Ensayos Clínicos del Instituto Nacional del Perú.

7.1.2. Invitación a participar, consentimiento informado, evaluación de elegibilidad y visita basal

El reclutador del estudio abordará a los pacientes que se encuentren en la zona de pre-triaje para atención de pacientes con síntomas respiratorios en los sites del estudio. A aquellos pacientes adultos se les preguntará cuántos días de síntomas tienen, y si son menos de 4 días (96 horas) y si han tomado ivermectina antes de asistir al hospital, si tienen historia de alergia a ivermectina o si toman algún otro medicamento. Aquellos que cumplen los criterios de inclusión serán invitados a participar en la preselección. Después de que firme el consentimiento de pre-selección se hará una evaluación de saturación de oxígeno con un pulsioxímetro, se evaluará la presencia de crepitantes, se tomará una prueba antigénica, una prueba serológica rápida y una prueba de embarazo en orina en caso de mujeres en edad reproductiva.

Si el paciente cumple los criterios de selección se hará el proceso de consentimiento informado para el ensayo y luego de las firmas se procederá a completar la visita basal.

En esta visita se llevarán a cabo los siguientes procedimientos:

- El personal de estudio completará la ficha de visita basal recogiendo datos demográficos, antecedentes médicos (hábitos nocivos, enfermedades previas) del participante y toda la medicación concomitante para confirmar la elegibilidad para participar en el ensayo.
- Tomará los datos antropométricos, funciones vitales del participante, y examen físico.
- Se tomará un hisopado nasofaríngeo para confirmar la presencia de ARN de SARS-CoV-2 (PCR).
- Se colectará una muestra de heces para examen coprológico basal. Si fuese el caso, la colección de esta primera muestra puede hacerse también al día siguiente en el domicilio del paciente. Se conoce que la presencia de helmintos podría tener un efecto inmunomodulador en su huésped en general. En el caso de la ivermectina, su efecto antiparasitario podría reducir ese efecto inmunomodulador, y en teoría podría condicionar una evolución más tórpida de la enfermedad [43]. Aún se sabe muy poco si la desparasitación por ivermectina podría además tener un efecto sobre el progreso de enfermedad COVID-19.
- Si el participante consintió el almacenamiento de muestras para análisis inmunológico posterior, se colectará una muestra de sangre de 10 ml. Esta muestra será congelada y almacenada en el Laboratorio de Enfermedades Tropicales del Hospital Nacional Cayetano Heredia, y será procesada en el mismo laboratorio cuando se consiga financiamiento.
- Se aleatorizará al paciente y se le administrará la primera dosis del producto en investigación dentro del site, de acuerdo con la tabla de dosificación por pesos. El personal del estudio supervisará la tolerancia inmediata durante 30 minutos.
- Si ocurriese inmediatamente algún EA o posteriormente, se registrará en la ficha de eventos adversos.
- A cada participante se le entregarán dos frascos con el PI asignado correspondientes a la segunda y tercera dosis para ser tomados en domicilio en día 2 y día 3. Un personal del estudio monitoreara la toma del PI por teléfono durante el día 2 y día 3.
- El personal del estudio dará instrucciones sobre la evaluación de síntomas diarios que tomará lugar desde el día 1 al día 21 del estudio. Esta evaluación diaria se llevará a cabo vía telefónica. .

7.1.3. Visitas subsecuentes de estudio

Las visitas subsecuentes se realizarán en el domicilio (del participante o donde se encuentre el paciente, en el caso que este hospitalizado) para evitar una exposición adicional a otras personas hasta obtener un resultado negativo de PCR en el hisopado nasofaríngeo, después del cual el paciente podrá decidir asistir a la atención ambulatoria.

En el estudio, se evaluarán diariamente los síntomas del participante a través de una llamada telefónica y la información se registrará en formato de evaluación diaria. Se seguirá a cada participante desde el día 2 al día 21.

A lo largo del estudio el médico visitará a los participantes en sus domicilios en los días 4, 7, 14 y 21 (5 veces) para realizar un examen físico, colección de muestras biológicas, y recopilación de nuevos medicamentos concomitantes, o eventos adversos.

En las visitas de estudio al domicilio en días 4, 7, 14 y 21:

- Un miembro del equipo del ensayo clínico preguntará al participante sobre la progresión de los síntomas y documentará la información en el formulario de visita de estudio.
- El médico realizará un examen físico y registrará los síntomas o signos en la ficha de seguimiento.
- Se realizará una prueba rápida serológica de SARS-CoV-2 para evaluar el estado inmunitario actual.
- Se tomará un hisopado nasofaríngeo para confirmar la presencia de ARN de SARS-CoV-2.
- El médico preguntará al participante si ha tomado algún medicamento o si ha buscado atención médica desde su última visita de estudio.
- Todas las muestras biológicas en estas visitas, excepto la prueba rápida, serán procesadas en el Laboratorio de Enfermedades Tropicales del Hospital Nacional Cayetano Heredia.

Procedimiento adicional en las visitas de estudio en días 7, 14 y 21:

Si el participante consintió el almacenamiento de muestras para análisis inmunológico posterior, se obtendrán y almacenarán cuatro muestras de sangre, de 10ml cada una, para estudios posteriores de inmunología. La extracción de sangre se llevará a cabo en las visitas de día basal, 7, 14 y 21. Los estudios inmunológicos serán:

- Células inmunes innatas (células dendríticas plasmacitoides y mieloides, células NK, macrófagos pro-inflamatorios, intermedios y clásicos) medidas en PBMC crio-preservado por flujo de citometría.
- Células CD4+ T y CD8+ T (% total de CD4+T y CD8+ T) expresando cualquier marcador funcional dentro de la simulación in vitro de PBMC con péptidos SARS-CoV-2, medidos por flujo de citometría.

La muestra de sangre se conservará a -20 ° C hasta por 5 años, en el Laboratorio de Enfermedades Tropicales del Hospital Nacional Cayetano Heredia. Se usará el código de investigación de participante para mantener la confidencialidad. No se realizarán estudios genéticos con las muestras de sangre almacenadas. Se solicitará la autorización para almacenamiento y uso futuro de muestras de sangre en consentimiento informado ampliado aparte. Si el participante retira su autorización, incluso después de terminada su participación en el estudio, estas muestras serán destruidas. Si estas muestras no se llegan a usar durante el periodo estimado, éstas serán destruidas. Estos estudios de inmunología se realizarán cuando se consiga financiamiento.

Procedimiento adicional en las visitas de estudio en días 14

- Se volverá a colectar una muestra de heces para estudio de helmintos intestinales mediante método de sedimentación espontánea en tubo descrita por Tello.

Procedimiento adicional para pacientes con muestras de hisopado nasofaríngeo basal positivo

- Se realizará cultivo viral de las muestras de hisopado nasofaríngeo de los pacientes que tuvieron un resultado positivo en la prueba molecular basal. El procedimiento será el siguiente:
  Aislamiento viral:
  Las muestras de hisopado nasofaríngeo serán filtradas en filtro con poro de 0,22 μm y alicuotadas en crioviales. Para el aislamiento viral será utilizada la línea celular VERO 81 cultivadas en medio de

cultivo DMEM (Dulbecco´s Modified Eagles Medium) suplementado con estreptomicina 100 mg/L, ampicilina 25 mg/L, 20% de suero fetal bovino (SFB) inactivado y mantenidas a 37 °C en atmosfera húmeda de 5% de CO_2. En frascos de 12.5 cc de superficie, se colocara una suspensión de células VERO y se dejara que forme una monocapa confluente, luego de un par de días se eliminar el medio de crecimiento e inoculará 200 µL de muestra de hisopado, incubar a 37 ˚C por 1 hora. Seguidamente será adicionado 3 mL de medio de mantenimiento celular al 2% de SBF. Las células serán observadas diariamente durante 10 días y se registrara la aparición de efecto citopático. La identificación del virus SARS-CoV-2 será realizada por IFI con anticuerpos específicos o RT-PCR en tiempo real.

Titulación viral:
Para conocer la carga del virus SARS-CoV-2 en las muestras (pre-tratamiento y pos-tratamiento) será utilizado el método de titulación en placas por contaje de las Unidades Formadoras de Placas (UFP). Diluciones (1/10, 1/100, 1/1000, 1/10000) de la muestra de hisopado serán inoculadas en células vero crecidas en placas de 24 pozos, e incubada por 1h a 37 ˚C, seguidamente 1 mL de medio overlay será adicionado y se incubara por 5 dias a 37 ˚C. Las placas de cultivo serán coloreadas con colorante cristal violeta y las placas de lisis viral serán cuantificadas.

7.2. Variables de valoración

Primaria

Proporción de pacientes con un resultado positivo de PCR para SARS-CoV-2 en un hisopo nasofaríngeo al día 7 después del tratamiento.

Secundarias

1. La media de la carga viral según lo determinado por el umbral del ciclo de PCR (Ct) en la visita de inscripción y los días 4, 7, 14 y 21.
2. Proporción de pacientes con fiebre y tos en los días 4, 7, 14 y 21.
3. Proporción de pacientes con seroconversión en el día 21.
4. Proporción de eventos adversos relacionados al producto en investigación durante el estudio.
5. Los niveles de IgG, IgM, IgA medidos por Luminex, las frecuencias de respuestas innatas y de células T evaluadas por citometría de flujo, los niveles de marcadores inflamatorios y de activación medidos por Luminex y transcriptómicos de los días 7, 14 y 21 (estos análisis se harán cuando se consiga financiamiento, a partir de las muestras de sangre congeladas y almacenadas en el Laboratorio de Enfermedades Tropicales del Hospital Nacional Cayetano Heredia).
6. Proporción y carga parasitaria de helmintos intestinales mediante método de sedimentación espontánea en tubo descrita por Tello de visita basal y día 14.

8. VALORACIÓN DE SEGURIDAD

Se evaluarán todos los eventos adversos reportados por los pacientes u observados por el equipo investigador, incluidas cualquier alteración analítica clínicamente relevante.

Se anotarán en la historia clínica del paciente cualquier EA que el paciente refiera espontáneamente o que aparezca como resultado de la anamnesis del investigador, especificando su fecha de inicio, evolución,

duración, intensidad, terapia requerida y relación con el medicamento en investigación. Se utilizarán los Criterios Comunes de Terminología Clínica para Eventos Adversos, versión 5.0 (CTCAE 5.0).

8.1. Definiciones

Evento adverso: definido como cualquier incidente que sea peligroso para la salud de un paciente o sujeto de un ensayo clínico tratado con un medicamento, incluso cuando no existe necesariamente una relación causal con dicha terapia.

Por lo tanto, un EA puede ser cualquier signo desfavorable e involuntario (incluido un hallazgo de laboratorio anormal), síntoma o enfermedad temporalmente asociada con el uso de un medicamento en investigación, independientemente de si está o no relacionado con el producto en investigación.

Evento adverso serio: definido como cualquier evento adverso o reacción adversa que, a cualquier dosis:

- Cause la muerte del paciente.
- Amenace la vida del paciente.
- Requiera que el paciente sea hospitalizado o que se extienda su hospitalización
- Cause invalidez o incapacidad permanente o mayor
- De lugar a una anomalía o deformación congénita

Para propósitos de notificación, cualquier sospecha de un evento adverso o reacción considerada como potencialmente importante desde un punto de vista médico será también considerada seria, incluso cuando no cumpla con los criterios anteriores, incluidos los eventos médicos importantes que requieren una intervención para prevenir que se produzca cualquiera de las consecuencias anteriormente mencionadas. Del mismo modo, también se reportarán como eventos adversos serios todas las sospechas de transmisión de un agente infeccioso a través del producto en investigación.

Reacción adversa (AR)

Se considera reacción adversa a cualquier reacción tóxica e involuntaria a un producto en investigación independientemente de la dosis administrada.

A diferencia de un EA, aquí sí existe una sospecha de relación causal entre el producto en investigación y el evento adverso.

La relación posible con el producto en investigación se establecerá de acuerdo con las siguientes definiciones:

Relación	Definición
NO RELACIONADO	No existe evidencia de alguna relación causal.
IMPROBABLE	Existe poca evidencia que sugiera una relación causal (p. ej. el evento no se presentó en un período de tiempo razonable posterior a la administración del producto / procedimiento en investigación). Existe otra explicación para el evento (p. ej. las condiciones clínicas del paciente, otras terapias concomitantes).
POSIBLE	Existe evidencia que sugiere una posible relación causal (p. ej. porque el evento ocurrió en un plazo razonable posterior a la administración del producto en investigación). Sin embargo, la influencia de otros factores puede haber contribuido al evento (p. ej. el estado clínico del paciente, otras terapias concomitantes).
PROBABLE	Existe evidencia que sugiere una relación causal y la influencia de otros factores es poco probable.
CIERTA	Existe evidencia clara que sugiere una relación causal y se puede descartar la posible contribución de otros factores.

Una reacción adversa debería considerarse como cualquier EA que esté posible, probable o definitivamente relacionado con el producto en investigación.

El investigador principal del centro de investigación, o la persona designada por el mismo, es responsable de establecer la posible relación causal con el producto en investigación.

Reacción adversa seria e inesperada (USAR)

Es cualquier reacción adversa seria cuya naturaleza, intensidad o consecuencias no coincidan con la información de referencia del medicamento (p. ej. el manual del investigador en el caso de un medicamento en investigación no autorizado o, el inserto en el caso de un medicamento autorizado).

La naturaleza inesperada de una reacción adversa está basada en el hecho que ésta no fue previamente observada y que no deberá basarse en lo que podría ser anticipada en vista de las propiedades farmacológicas del medicamento.

8.2. Registro y comunicación de eventos adversos

Todos los eventos adversos notificados serán registrados, ya sea espontáneamente por los pacientes o durante las entrevistas mantenidas con ellos en las visitas de estudio. Se registrarán los eventos adversos desde que el paciente recibió la primera dosis del producto en investigación hasta, el final de su participación en el estudio.

Se solicitará al paciente que facilite toda la información sobre los eventos adversos ocurridos en el período de tiempo comprendido entre la visita anterior y la actual.

Todos los eventos adversos deberán ser documentados en la historia clínica del paciente y en la "Ficha de Eventos Adversos".

Cuando el investigador considere que un evento adverso es serio, deberá notificarlo inmediatamente al patrocinador tan pronto como tenga conocimiento de ello.

8.3. Eventos adversos serios que no requieren comunicación a la persona responsable de farmacovigilancia

El investigador deberá notificar inmediatamente todos los EAS, según las definiciones anteriormente mencionadas, con la excepción de los siguientes eventos adversos serios:

- EAS que se produzcan después de 5 días de tratamiento (ya que la vida media de la ivermectina es de 17 horas)
- Hospitalización o muerte debido a la progresión de la enfermedad

Todas las EAS se registrarán en la historia clínica del paciente y en el CRF.

Los demás EAS deberán ser notificados según el procedimiento descrito en el punto 8.4.

8.4. Procedimiento de notificación de EAS

Si existe un EAS que deba ser notificado a la persona responsable de farmacovigilancia, un miembro del equipo de investigación completará y firmará el formulario de reporte de EAS, el cual deberá ser enviado escaneado por correo electrónico, inmediatamente y siempre dentro de las 24 horas siguientes al momento en que se conoció el hecho.

La persona responsable de farmacovigilancia comprobará el formulario recibido y, si es necesario, solicitará al investigador información adicional.

En el momento en que se obtenga información adicional sobre el EAS, o en caso de que la situación se resuelva o de que sea improbable que cambie, se deberá cumplimentar un reporte de vigilancia y enviarlo escaneado por correo electrónico a la persona responsable de farmacovigilancia.

Si se sospecha que el EAS pueda ser una SUSAR, el investigador deberá proporcionar toda la información de vigilancia que pueda ser solicitada.

Cualquier EAS que se descubra y ocurra durante el primer mes posterior a la finalización del ensayo deberá ser notificado (sin límites de tiempo), si el investigador considera que está relacionado con el producto en investigación (p.ej. si es una reacción adversa seria) o si es médicamente importante.

Embarazo

Todas las mujeres en edad reproductiva se someterán a una prueba de embarazo en orina para confirmar los criterios de inclusión/exclusión. Aquellas mujeres en edad reproductiva que consientan participar en el estudio recibirán consejería sobre la importancia del evitar el embarazo mientras dure su participación en el estudio. En el caso de que la participante no use un método anticonceptivo al momento de ingresar al estudio, nosotros brindaremos un método sin costo durante la duración del estudio, elegido por la participante y que no sea incompatible con el ensayo clínico.

Durante las campañas comunitarias de ivermectina para oncocercosis, con dosis inferiores a la aprobada por la EMA, se excluyen a las mujeres visiblemente embarazadas pero no se realiza ninguna prueba de embarazo de manera rutinaria a mujeres en edad reproductiva. Existen varios reportes de tratamiento inadvertido con ivermectina durante el embarazo, en un metanálisis reciente por Nicolas et al [37], se

analizaron datos de 893 embarazos expuestos a ivermectina de forma inadvertida (97 en el primer trimestre) y se concluyó que no hay suficiente evidencia para entender la seguridad de ivermectina durante el embarazo.

8.5. Interrupción del producto en investigación debido a eventos adversos

Se interrumpirán las subsecuentes dosis si se presentase algún evento que ponga el riesgo la integridad del participante o sea considerado como relevante por el médico de estudio.

9. Análisis estadístico

9.1. Descripción de los métodos estadísticos a ser usados

Se describirán los datos de basales de los pacientes inscritos en ambos brazos de ensayo. En este análisis descriptivo se utilizarán la frecuencia, la mediana y el rango intercuartil para las variables cualitativas y cuantitativas respectivamente.

Todos los análisis se harán utilizando los programas STATA y R.

EVALUACIÓN DE LA EFICACIA

La principal variable de valoración se evaluará utilizando la prueba exacta de Fisher. Esto permitirá comparar la proporción de participantes con resultado positivo de PCR al séptimo día después del tratamiento.

El análisis se hará como indicado en el protocolo, incluyendo a todos los participantes aleatorizados para los que haya un resultado de PCR al día siete. Los pacientes aleatorizados que no tengan resultados de PCR del día siete serán descritos para verificar no haya un patrón común.

EVALUACIÓN DE SEGURIDAD

El análisis de seguridad se hará utilizando un análisis modificado por intención de tratar considerando a todos los participantes aleatorizados. Se describirán por separado los EA experimentados por los participantes de cada brazo de estudio

Todos los eventos adversos que ocurran durante el estudio se incluirán en las listas de datos y se organizarán de acuerdo a cada paciente. Los eventos que se consideren relacionados con el producto en investigación (posible, probable o definitivamente relacionados) también se incluirán en una tabla. Se proveerá una tabla que enlista los eventos adversos según su máxima intensidad. Las muertes y los eventos adversos serios también se clasificarán en una tabla aparte.

9.2. *Número de sujetos planeados a ser incluidos y poder del estudio*

El tamaño de la muestra para nuestro estudio ha sido calculado para comparar dos proporciones. Una muestra de 186 participantes con PCR positivo (93 por brazo) proporciona un 80% de poder al 5% de nivel de significancia para detectar una diferencia de al menos 10% (90% en el grupo de intervención vs. 100% en el grupo control) en la proporción de participantes con resultado positivo de PCR al día 7 de tratamiento. Se estima un 20% de pérdidas al seguimiento o resultados falsos positivos en la prueba de antígeno, por lo que se requiere aleatorizar 236 participantes (118 en cada brazo) para el ensayo clínico. Estos participantes serán divididos en 59 bloques completos aleatorizados de tamaño 4. Se espera que en

la fase de pre-selección una de cada 8 personas potencialmente elegibles tenga un resultado positivo en la prueba de antígeno y cumpla los otros criterios, por lo que se deberá incluir 1872 personas en esta fase.

9.3. Nivel de significancia a ser usado

El nivel de significancia estadística ha sido establecido en 5%

9.4. Criterios para terminar el estudio

El estudio normalmente terminará cuando se haya inscrito el número planeado de pacientes o no se puedan reclutar más y el último paciente haya completado el ensayo y se hayan resuelto todas las inconsistencias y los eventos adversos.

La razón por la cual podría ser necesario terminar el estudio:

- La incidencia y/o gravedad de los eventos adversos en este u otros estudios indica un posible riesgo para la salud por el tratamiento con el producto en investigación o de referencia.

En todos estos casos se deberá realizar una evaluación final a cada paciente que permanezca en el estudio en el momento en se suspenda o se termine el estudio anticipadamente. El investigador deberá completar los formularios para notificación de casos (CRF) con el mayor detalle posible.

9.5. Procedimiento usado para contabilizar los datos perdidos, no utilizados e incorrectos

Todos los datos disponibles sobre la seguridad y eficacia se incluirán en las listas y tablas de datos. No se asignarán valores a los datos no disponibles.

Todo dato confusor o incorrecto deberá ser examinado en conformidad con los procedimientos estándar de control de datos.

En el análisis de los registros notificados por los pacientes, los datos omitidos se asignarán utilizando la última observación realizada. La norma omitida se examinará antes de cualquier asignación. Si el patrón omitido es claramente informativo, la repercusión de los datos no aleatorios se evaluará mediante un análisis de sensibilidad.

Para el análisis de los registros notificados por pacientes, si falta una sección de la subescala de varias, se utilizará el promedio de las secciones restantes como escala de puntuación, siempre que se disponga al menos de la mitad de las secciones de la escala.

9.6. Procedimiento para informar de las desviaciones del plan estadístico original

Toda desviación del plan de análisis estadístico original se incluirá en el reporte final del ensayo clínico.

9.7. Selección de los sujetos que se incluirán en cada análisis

El análisis de eficacia se hará por protocolo, incluyendo a todos los participantes aleatorizados para los que haya un resultado de PCR al día siete.

El análisis de seguridad se hará utilizando un análisis modificado por intención de tratar considerando a todos los participantes aleatorizados y tratados.

10. ASPECTOS ÉTICOS

10.1. Buenas prácticas clínicas

El estudio se llevará a cabo en conformidad con la Conferencia Internacional de Armonización (ICH) en lo que respecta a la Buenas Prácticas Clínicas y los requisitos reglamentarios correspondientes. El investigador deberá estar completamente familiarizado con el uso correcto del producto en investigación tal y como se describe en el protocolo. Se conservarán los documentos clínicos esenciales para demostrar la validez del estudio y la integridad de los datos recogidos. Los archivos principales serán creados al inicio del estudio, conservados durante el transcurso del mismo, y almacenados de acuerdo con la legislación pertinente.

10.2. Consideraciones éticas

El estudio se llevará a cabo de acuerdo con los principios éticos de la última revisión de la Declaración de Helsinki y con la legislación vigente. El CNTEI-COVID19 examinará toda la documentación relacionada al estudio para proteger los derechos, la seguridad y el bienestar de los pacientes. El estudio sólo se llevará a cabo en los centros para los que se haya obtenido la aprobación del CNTEI. El protocolo se presentará al CNTEI, junto con el consentimiento informado, la publicidad, la información escrita dada a los pacientes, las actualizaciones relacionadas con la seguridad, los informes anuales de progreso y cualquier cambio realizado en los documentos anteriores.

Este estudio sólo podrá comenzar después de que se reciba una decisión favorable por escrito del CNTEI-COVID19 y de que la OGITT dé su aprobación.

10.3. Información para el paciente y consentimiento informado

El paciente recibirá el Formulario de consentimiento informado y se le informará que la participación en el estudio es voluntaria y puede retirarse en cualquier momento sin perjuicio de la atención médica previa. No se puede modificar ni la Hoja informativa para el paciente ni el Consentimiento informado sin el acuerdo del CNTEI y el patrocinador.

Durante este período de pandemia de SARS-CoV-2, no se conservarán los formato de consentimiento informado original (FCI). En su lugar se tomarán fotografías para los registros del investigador. Este consentimiento informado se obtendrá una vez se expliquen los detalles del estudio en su totalidad al paciente, y sólo después se procederá a hacer efectiva su participación en el estudio. El investigador debe informar completamente al paciente sobre lo que está consintiendo y se obtendrá la firma del consentimiento informado. El investigador (o la persona delegada por el mismo) deberá firmar y fechar el formulario de consentimiento. Esto es para el consentimiento de pre-selección y para el consentimiento del ensayo clínico.

Se obtendrá además un segundo consentimiento sobre el almacenamiento de muestra para uso futuro. El participante puede elegir si autorizar o no, sin alterar su participación. Al igual que los formatos de consentimiento informado principal, este formato de consentimiento de almacenamiento de muestras no será archivado en el site. En su lugar, se tomará una fotografía para los records del investigador, y se entregará el formato original firmado al participante.

El formulario de consentimiento incluye información sobre la necesidad de inspeccionar las historias clínicas y permitir la obtención de datos básicos en caso de que el paciente decida suspender su participación por razones diferentes a la retirada del consentimiento.

10.4. Confidencialidad de pacientes

Con el fin de respetar la privacidad de los pacientes, se les identificará por medio de un número asignado a cada participante en todos los formularios para notificación de casos, registros de responsabilidad de producto en investigación, informes y comunicados del estudio. El investigador proporcionará a los inspectores y a los posibles auditores o colaboradores designados por el patrocinador y las autoridades reguladoras el acceso a los registros originales de los pacientes para que puedan verificar los datos en los formularios para notificación de casos y auditar el proceso de recopilación de datos. Se mantendrá la confidencialidad y la identidad del paciente no se hará pública, en la medida permitida por la legislación y los reglamentos pertinentes.

10.5. Compensación para el paciente

No habrá compensación al paciente por participar en el estudio

10.6. Póliza de seguros

El patrocinador del estudio es la Universidad Peruana Cayetano Heredia, y su representante legal, el Dr. Alejandro Busalleu. La póliza se comprará una vez que se tenga la aprobación respectiva, como se ha hecho en otros ensayos. No se comenzará a reclutar hasta haber enviado la póliza al INS.

10.7. Terminación anticipada de estudio

Este estudio podría ser interrumpido anticipadamente si el patrocinador o las autoridades reguladoras consideran que existe una causa suficiente para hacerlo. El investigador recibirá una notificación por escrito en la que la parte que transfiere documenta el motivo para la suspensión del estudio.

Las circunstancias que pueden justificar la suspensión del estudio incluyen, pero no se limitan a:

- Establecimiento de riesgos inesperados que sean considerables o inaceptables para los pacientes.
- Número insuficiente de pacientes inscritos.
- Cumplimiento insuficiente de los requisitos del protocolo.

La razón por la cual puede ser necesario terminar el estudio:

- La incidencia y/o la gravedad de los eventos adversos en este u otros estudios que indiquen un posible riesgo para la salud por el tratamiento con el producto en investigación o de referencia.

En todos estos casos, se debe realizar una evaluación final a cada paciente que se mantengan en el estudio al momento de cierre o terminación anticipada. El investigador completará los formularios para notificación de casos con el mayor detalle posible. El patrocinador recogerá los formularios para notificación de casos completados así como los materiales de estudio.

11. GESTIÓN DE DATOS

11.1. Formularios para Notificación de Casos (ANEXOS 3 al 8 CRF)

Los CRF serán todos llenados de manera electrónica utilizando un sistema de registro de datos en tablets con el programa Magpie y otros.

Cualquier modificación en la base de datos se registrará en un registro de modificaciones, junto con la información correspondiente de por qué se realizó el cambio, quién lo hizo y la fecha de modificación. Al

finalizar el estudio, un CD-ROM cifrado que almacena los CRF digitales será generado para ser archivado. Cualquier resultado fuera del rango de normalidad será debidamente comentado.

11.2. Reporte final y Publicación

El equipo de investigación elaborará un reporte adecuado para su presentación a las autoridades pertinentes. Se elaborará al menos un manuscrito para presentar los resultados, independientemente de si éstos son negativos o positivos.

12. REFERENCIAS

1. Richards FO, Jr.: Upon entering an age of global ivermectin-based integrated mass drug administration for neglected tropical diseases and malaria. Malar J 2017, 16:168.
2. Frieman M, Yount B, Heise M, Kopecky-Bromberg SA, Palese P, Baric RS: Severe Acute Respiratory Syndrome Coronavirus ORF6 Antagonizes STAT1 Function by Sequestering Nuclear Import Factors on the Rough Endoplasmic Reticulum/Golgi Membrane. Journal of Virology 2007, 81:9812-24.
3. Zhou Y, Hou Y, Shen J, Huang Y, Martin W, Cheng F: Network-based drug repurposing for novel coronavirus 2019-nCoV/SARS-CoV-2. Cell Discovery 2020, 6:14.
4. Jang K-J, Jeong S, Kang DY, Sp N, Yang YM, Kim D-E: A high ATP concentration enhances the cooperative translocation of the SARS coronavirus helicase nsP13 in the unwinding of duplex RNA. Scientific Reports 2020, 10:4481.
5. Adedeji AO, Marchand B, Te Velthuis AJ, Snijder EJ, Weiss S, Eoff RL et al: Mechanism of nucleic acid unwinding by SARS-CoV helicase. PLoS One 2012, 7:e36521.
6. Mehta P, McAuley DF, Brown M, Sanchez E, Tattersall RS, Manson JJ et al: COVID-19: consider cytokine storm syndromes and immunosuppression. Lancet 2020.
7. Huang C, Wang Y, Li X, Ren L, Zhao J, Hu Y et al: Clinical features of patients infected with 2019 novel coronavirus in Wuhan, China. Lancet 2020, 395:497-506.
8. Omura S, Crump A: Ivermectin: panacea for resource-poor communities? Trends Parasitol 2014, 30:445-55.
9. Tay MY, Fraser JE, Chan WK, Moreland NJ, Rathore AP, Wang C et al: Nuclear localization of dengue virus (DENV) 1-4 non-structural protein 5; protection against all 4 DENV serotypes by the inhibitor Ivermectin. Antiviral Res 2013, 99:301-6.
10. Yang SNY, Atkinson SC, Wang C, Lee A, Bogoyevitch MA, Borg NA et al: The broad spectrum antiviral ivermectin targets the host nuclear transport importin alpha/beta1 heterodimer. Antiviral Res 2020:104760.
11. Wagstaff KM, Sivakumaran H, Heaton SM, Harrich D, Jans DA: Ivermectin is a specific inhibitor of importin alpha/beta-mediated nuclear import able to inhibit replication of HIV-1 and dengue virus. Biochem J 2012, 443:851-6.
12. Barrows NJ, Campos RK, Powell ST, Prasanth KR, Schott-Lerner G, Soto-Acosta R et al: A Screen of FDA-Approved Drugs for Inhibitors of Zika Virus Infection. Cell Host Microbe 2016, 20:259-70.
13. Varghese FS, Kaukinen P, Glasker S, Bespalov M, Hanski L, Wennerberg K et al: Discovery of berberine, abamectin and ivermectin as antivirals against chikungunya and other alphaviruses. Antiviral Res 2016, 126:117-24.
14. Mastrangelo E, Pezzullo M, De Burghgraeve T, Kaptein S, Pastorino B, Dallmeier K et al: Ivermectin is a potent inhibitor of flavivirus replication specifically targeting NS3 helicase activity: new prospects for an old drug. J Antimicrob Chemother 2012, 67:1884-94.
15. Lundberg L, Pinkham C, Baer A, Amaya M, Narayanan A, Wagstaff KM et al: Nuclear import and export inhibitors alter capsid protein distribution in mammalian cells and reduce Venezuelan Equine Encephalitis Virus replication. Antiviral Res 2013, 100:662-72.
16. Lee YJ, Lee C: Ivermectin inhibits porcine reproductive and respiratory syndrome virus in cultured porcine alveolar macrophages. Arch Virol 2016, 161:257-68.
17. Caly L, Druce J, Catton M, Jans D, KM W: The FDA-approved Drug Ivermectin inhibits the replication of SARS-CoV-2 in vitro. Antiviral Research 2020, In Press, Journal Pre-proof.
18. Yamasmith, E; et al. Efficacy and Safety of Ivermectin against Dengue Infection: A Phase III, Randomized, Double-blind, Placebo-controlled Trial, in he 34th Annual Meeting The Royal College of Physicians of Thailand- 'Internal Medicine and One Health'. 2018: Chonburi, Thailand. Registry available at: https://clinicaltrials.gov/ct2/show/NCT02045069 (accessed Aplri 5, 2020).

19. Lespine A, Alvinerie M, Sutra JF, Pors I, Chartier C: Influence of the route of administration on efficacy and tissue distribution of ivermectin in goat. Vet Parasitol 2005, 128:251-60.
20. Chiu SH, Lu AY: Metabolism and Tissue Residues. In: WC Campbell (ed),Ivermectin and Abamectin. New York: Springer-Verlag; 1989: 131–43.
21. Zhang X, Song Y, Ci X, An N, Ju Y, Li H et al: Ivermectin inhibits LPS-induced production of inflammatory cytokines and improves LPS-induced survival in mice. Inflamm Res 2008, 57:524-9.
22. Ci X, Li H, Yu Q, Zhang X, Yu L, Chen N et al: Avermectin exerts anti-inflammatory effect by downregulating the nuclear transcription factor kappa-B and mitogen-activated protein kinase activation pathway. Fundam Clin Pharmacol 2009, 23:449-55.
23. Blakley BR, Rousseaux CG: Effect of ivermectin on the immune response in mice. Am J Vet Res 1991, 52:593-5.
24. Ministerio de salud. Resolución Ministerial N° 270-2020-MINSA. [Internet]. [citado en junio 2020]. Disponible en: https://www.gob.pe/institucion/minsa/normas-legales/563764-270-2020-minsa
25. Ministerio de salud. Resolución Ministerial N° 375-2020-MINSA. [Internet]. [citado en junio 2020]. Disponible en: https://cdn.www.gob.pe/uploads/document/file/829755/RM_375-2020-MINSA.PDF
26. U.S. National Library of Medicine. ClinivalTrials.gov. Albendazol Plus High Dose Ivermectin for Trichuriasis in Pediatric Patients. (NCT04041453). [Internet]. [citado en junio 2020]. Disponible en: https://clinicaltrials.gov/ct2/show/record/NCT04041453
27. Gonzalez Canga A, Sahagun Prieto AM, Diez Liebana MJ, Fernandez Martinez N, Sierra Vega M, Garcia Vieitez JJ: The pharmacokinetics and interactions of ivermectin in humans--a mini-review. AAPS J 2008, 10:42-6.
28. Merck_&_Co.: Stromectrol. FDA approved Package insert 2009. http://www.accessdata.fda.gov/drugsatfda_docs/label/2009/050742s026lbl.pdf .
29. Merck_&_Co.: Stromectrol. TGA-Australia approved Package insert 2014. https://www.ebs.tga.gov.au/ebs/picmi/picmirepository.nsf/pdf?OpenAgent&id=CP-2011-PI-02659-3&d=2016071016114622483.
30. Kamgno J, Gardon J, Gardon-Wendel N, Demanga N, Duke BO, Boussinesq M: Adverse systemic reactions to treatment of onchocerciasis with ivermectin at normal and high doses given annually or three-monthly. Trans R Soc Trop Med Hyg 2004, 98:496-504.
31. Smit MR, Ochomo EO, Aljayyoussi G, Kwambai TK, Abong'o BO, Chen T et al: Safety and mosquitocidal efficacy of high-dose ivermectin when co-administered with dihydroartemisinin-piperaquine in Kenyan adults with uncomplicated malaria (IVERMAL): a randomised, double-blind, placebo-controlled trial. Lancet Infect Dis 2018, 18:615-26.
32. Awadzi K, Opoku NO, Addy ET, Quartey BT: The chemotherapy of onchocerciasis. XIX: The clinical and laboratory tolerance of high dose ivermectin. Trop Med Parasitol 1995, 46:131-7.
33. Awadzi K, Attah SK, Addy ET, Opoku NO, Quartey BT: The effects of high-dose ivermectin regimens on Onchocerca volvulus in onchocerciasis patients. Trans R Soc Trop Med Hyg 1999, 93:189-94.
34. Guzzo CA, Furtek CI, Porras AG, Chen C, Tipping R, Clineschmidt CM et al: Safety, tolerability, and pharmacokinetics of escalating high doses of ivermectin in healthy adult subjects. J Clin Pharmacol 2002, 42:1122-33.
35. Munoz J, Ballester MR, Antonijoan RM, Gich I, Rodriguez M, Colli E et al: Safety and pharmacokinetic profile of fixed-dose ivermectin with an innovative 18mg tablet in healthy adult volunteers. PLoS Negl Trop Dis 2018, 12:e0006020.
36. Gardon J, Gardon-Wendel N, Demanga N, Kamgno J, Chippaux JP, Boussinesq M: Serious reactions after mass treatment of onchocerciasis with ivermectin in an area endemic for Loa loa infection. Lancet 1997, 350:18-22.
37. Nicolas P, Maia MF, Bassat Q, Kobylinski KC, Monteiro W, Rabinovich NR et al: Safety of oral ivermectin during pregnancy: a systematic review and meta-analysis. Lancet Glob Health 2020, 8:e92-e100.

38. Wolfel R, Corman VM, Guggemos W, Seilmaier M, Zange S, Muller MA et al: Virological assessment of hospitalized patients with COVID-2019. Nature 2020.
39. He X, Lau EHY, Wu P, Deng X, Wang J, Hao X et al: Temporal dynamics in viral shedding and transmissibility of COVID-19. Nat Med 2020.
40. To KK, Tsang OT, Leung WS, Tam AR, Wu TC, Lung DC et al: Temporal profiles of viral load in posterior oropharyngeal saliva samples and serum antibody responses during infection by SARS-CoV-2: an observational cohort study. Lancet Infect Dis 2020.
41. Zou L, Ruan F, Huang M, Liang L, Huang H, Hong Z et al: SARS-CoV-2 Viral Load in Upper Respiratory Specimens of Infected Patients. N Engl J Med 2020, 382:1177-79.
42. Zhou F, Yu T, Du R, Fan G, Liu Y, Liu Z et al: Clinical course and risk factors for mortality of adult inpatients with COVID-19 in Wuhan, China: a retrospective cohort study. Lancet 2020, 395:1054-62.
43. Bradbury, R. S., Piedrafita, D., Greenhill, A., & Mahanty, S. (2020). Will helminth co-infection modulate COVID-19 severity in endemic regions?. Nature Reviews Immunology, 20(6), 342-342.
44. Carlos Chaccour, Aina Casellas, Andres Blanco-Di Matteo et al. The effect of early treatment with ivermectin on viral load, symptoms and humoral response in patients with mild COVID-19: a pilot, double-blind, placebo-controlled, randomized clinical trial, 07 December 2020, PREPRINT (Version 1) available at Research Square [https://doi.org/10.21203/rs.3.rs-116547/v1]

ANEXO 1: TABLA DE DOSIFICACIÓN DISCRETA

Dosis deseada 300 mcg/kg			
**Peso**	**dosis total (mg)**	**ml**	**gotas**
**45**	13.5	2.3	68
**46**	13.8	2.3	69
**47**	14.1	2.4	71
**48**	14.4	2.4	72
**49**	14.7	2.5	74
**50**	15	2.5	75
**51**	15.3	2.6	77
**52**	15.6	2.6	78
**53**	15.9	2.7	80
**54**	16.2	2.7	81
**55**	16.5	2.8	83
**56**	16.8	2.8	84
**57**	17.1	2.9	86
**58**	17.4	2.9	87
**59**	17.7	3.0	89
**60**	18	3.0	90
**61**	18.3	3.1	92
**62**	18.6	3.1	93
**63**	18.9	3.2	95
**64**	19.2	3.2	96
**65**	19.5	3.3	98
**66**	19.8	3.3	99
**67**	20.1	3.4	101
**68**	20.4	3.4	102
**69**	20.7	3.5	104
**70**	21	3.5	105
**71**	21.3	3.6	107
**72**	21.6	3.6	108
**73**	21.9	3.7	110
**74**	22.2	3.7	111
**75**	22.5	3.8	113
**76**	22.8	3.8	114
**77**	23.1	3.9	116
**78**	23.4	3.9	117
**79**	23.7	4.0	119
**80**	24	4.0	120
**81**	24.3	4.1	122

**82**	24.6	4.1	123
**83**	24.9	4.2	125
**84**	25.2	4.2	126
**85**	25.5	4.3	128
**86**	25.8	4.3	129
**87**	26.1	4.4	131
**88**	26.4	4.4	132
**89**	26.7	4.5	134
**90**	27	4.5	135
**91**	27.3	4.6	137
**92**	27.6	4.6	138
**93**	27.9	4.7	140
**94**	28.2	4.7	141
**95**	28.5	4.8	143
**96**	28.8	4.8	144
**97**	29.1	4.9	146
**98**	29.4	4.9	147
**99**	29.7	5.0	149
**100**	30	5.0	150
**101**	30.3	5.1	152
**102**	30.6	5.1	153
**103**	30.9	5.2	155
**104**	31.2	5.2	156
**105**	31.5	5.3	158
**106**	31.8	5.3	159
**107**	32.1	5.4	161
**108**	32.4	5.4	162
**109**	32.7	5.5	164
**110**	33	5.5	165
**111**	33.3	5.6	167
**112**	33.6	5.6	168
**113**	33.9	5.7	170
**114**	34.2	5.7	171
**115**	34.5	5.8	173
**116**	34.8	5.8	174
**117**	35.1	5.9	176
**118**	35.4	5.9	177
**119**	35.7	6.0	179
**120**	36	6.0	180

ANEXO 2: Consentimiento Informado de Pre-selección

FORMULARIO DE CONSENTIMIENTO INFORMADO PARA LA PRE-SELECCIÓN PARTICIPAR EN UN ESTUDIO CLÍNICO (V1.0)

CONSENTIMIENTO INFORMADO PARA PRESELECCION	
**Título:**	Ensayo Clínico aleatorizado de Fase IIa para comparar la efectividad de la ivermectina versus placebo en la negativización del PCR en pacientes en fase temprana de COVID-19
**Investigadores:**	Dra. Patricia García, Dr. Jesús Chacaltana Dr. Carlos Chaccour, Dr. César Cárcamo, Dra. Patricia León, Dr. Germán Málaga, Dr. Hansel Mundaca, Dr. César Ugarte
**Patrocinador:**	Universidad Peruana Cayetano Heredia
**Institucion:**	Hospital Nacional Cayetano Heredia y Hospital Daniel Alcides Carrión

INTRODUCCION

Se le está invitando a participar en la pre-selección para una investigación clínica, dirigida por la Universidad Cayetano Heredia, que ha sido aprobada por el Comité Nacional Transitorio de Ética en Investigación.

Es importante que entienda la diferencia entre la atención regular que obtiene de su médico y la que está relacionada a esta pre-selección para el estudio. El fin de la atención médica es monitorizar y mejorar su salud. El fin de un estudio de investigación es recabar información. Su participación en este estudio es totalmente voluntaria, nadie lo puede obligar a participar; puede decidir participar ahora y cambiar de opinión posteriormente.

Si decide participar en esta pre-selección al estudio, se le pedirá que firme este documento de consentimiento antes de que se realice cualquier actividad relacionada al estudio. Recibirá una copia de este documento. Si ud. es seleccionado se le pedirá luego que firme el consentimiento para participar en el estudio.

¿Cuál es el propósito del estudio de investigación?

Al momento no existe un tratamiento específico aprobado contra el COVID-19. En este estudio de investigación queremos evaluar si la ivermectina realmente puede tener actividad contra el virus del COVID-19. Para esto algunos de los participantes al azar recibirán Ivermectina y otros recibirán solo un placebo. El placebo es un compuesto farmacológico que no contiene ivermectina, pero que al tomarla le será indistinguible en sabor y apariencia. Ni siquiera el médico que lo trata sabrá si se le dio ivermectina o placebo. A todos los pacientes se les hará un seguimiento diario, y visitas domiciliarias. También se tomarán pruebas de sangre y moleculares para ver el efecto si hay o no efecto de la ivermectina.

¿Cuántas personas participarán en este pre-selección?

Se invitará a la pre-selección hasta 1872 participantes mayores de edad diagnosticados con COVID-19 leve y con menos de 96 horas de inicio de síntomas típicos (fiebre, tos, dolor de cabeza, etc.). Los pacientes serán reclutados al acudir al Hospital Cayetano Heredia y Hospital Daniel Alcides Carrión.

¿Por qué me han invitado?

Se le invita a participar en esta pre-selección al estudio debido a que usted presenta un cuadro clínico sospechoso de de COVID-19. Además, porque Ud. es mayor de 18 años, ha tenido síntomas de COVID-19 por menos de 96 horas, y que no ha tomado ivermectina anteriormente en cualquiera de sus presentaciones.

¿Cuánto tiempo durará mi participación en la pre-selección ?

La duración de su participación la pre-selección será solo lo que dure el examen clínico y la toma de muestras. Si ud es elegible, se le invitará a participar en el estudio, luego de firmar otro consentimiento y su participación en el estudio durará 21 días. Iniciará con el examen aquí en hospital, y lo seguiremos monitoreando y se le visitará en su domicilio o donde se encuentre, los días 4, 7, 14, 21.

¿Qué involucra esta pre-selección?

Si Ud. decide participar, luego de haber respondido todas sus preguntas y haber firmado este consentimiento, Ud. pasará a ser visto por el médico del estudio quien le medirá la cantidad de oxígeno en la sangre y escuchará sus pulmones, si hay alguna sospecha de posible neumonía, lo referirá al servicio del hospital y no será reclutado en el

estudio. Si todo está bien, se le tomará una muestra de sangre para realizar una prueba rápida de serología contra el COVID-19 y una muestra nasofaríngea para una prueba de antígeno para detectar el virus, y de acuerdo a estos resultados se le referirá o Ud será invitado al participar en el estudio y se le pedirá que firme un consentimiento informado de participación en el estudio. Si usted es mujer en edad reproductiva, se colectará una muestra de orina para descartar embarazo, puesto que no participarán mujeres embarazadas en el estudio.

¿Cuáles son riesgos y potenciales beneficios de participar en el pre-screening?

La extracción de sangre tiene un pequeño riesgo de infección y puede ser un poco dolorosa. Su brazo podría tener algún hematoma durante algunos días. La toma de muestra nasofaringea puede ser un poco incómoda. El beneficio es que contará con resultados de pruebas sobre su enfermedad.

Costos y pago

No hay costos por su participación en el estudio. Usted no tendrá que pagar por ningún medicamento ni por ninguna prueba del estudio. Usted no recibirá pago por participar en el estudio.

Confidencialidad

Usted será identificado con un código. Garantizamos mantener su privacidad de toda su información tal y como lo exige la ley. Su información médica y del estudio podrá ser revisada solo por el equipo del estudio, el CNTEI, y el Instituto Nacional de Salud del Perú. Los investigadores del centro se asegurarán de que cualquier persona que no esté relacionada con el estudio no tenga acceso a sus datos del estudio.

Derechos de los participantes

Al dar su consentimiento para participar en este pre-selección, usted no renuncia a ninguno de sus derechos legales. Dar consentimiento significa que usted ha escuchado o leído la información y que está de acuerdo en participar. Usted recibirá el formato original para sus registros personales. Si en algún momento usted se retira no sufrirá penalización alguna ni perderá ningún beneficio al que tiene derecho. Toda información este momento continuará siendo parte del estudio. Le daremos a conocer cualquier información nueva que pueda afectar su salud, bienestar o voluntad de permanecer en este estudio. Si usted quiere conocer los resultados del estudio, hágaselo saber al personal del estudio.

Compensación de lesiones relacionadas a investigación

Si usted sufre un daño o lesión relacionado directamente con el estudio, se le derivará para un tratamiento inmediato y gratuito a través del SIS, usted no tendrá que pagar por esta atención. No existe ningún otro programa de compensación. Usted no renuncia a ninguno de sus derechos legales al firmar este formulario de consentimiento.

PROBLEMAS O PREGUNTAS

Este estudio ha sido revisado y aprobado por el Comité Nacional Transitorio de Ética en Investigación para la evaluación y supervisión éticas de los Ensayos Clínicos de la enfermedad COVID-19 (CNTEI-COVID-19) para asegurarse de que los participantes de la investigación estén protegidos. Si usted tiene preguntas sobre sus derechos como participante, puede comunicarse al correo electrónico: comite_etica_covid19@ins.gob.pe y/o con el Dr. Aldo Vivar, Presidente del CNTEICovid-19, al teléfono: 992705648.

a) Contactos para responder cualquier duda o pregunta y en caso de lesiones - Investigadora Principal:
Dra. Patricia García por correo a patricia.garcia@upch.pe, o investigador Dr. Hansel Mundaca al teléfono 989 675 386 o escribiendo al correo electrónico hansel.mundaca@upch.pe.

b) Datos de contacto de la Autoridad Reguladora (INS). "Cuando usted considere que sus derechos son vulnerados o ante cualquier denuncia, usted puede contactarse con el INS (Oficina General de Investigación y Transferencia Tecnológica, OGITT), entidad reguladora de ensayos clínicos, a través del siguiente teléfono: 7481111 anexo 2191 o mediante comunicación escrita a través del siguiente correo electrónico: consultaensayos@ins.gob. pe, o mediante un documento formal presentado a través de mesa de partes de la institución o acudir en persona a la OGITT en la siguiente dirección: Cápac Yupanqui 1400, Jesús María, Lima 11".

Consentimiento Informado para preselección

TÍTULO DEL ESTUDIO: Ensayo Clínico aleatorizado de Fase IIa para comparar la efectividad de la ivermectina versus placebo en la negativización del PCR en pacientes en fase temprana de COVID-19

**Codigo de pre-selección:**	
Leí la información, o me fue leída. Pude hacer cualquier pregunta que quisiera, y recibí respuestas satisfactorias a mis preguntas	
	Doy mi consentimiento para participar en este estudio.
**Nombre y apellido**	
**Firma**	
**Fecha**	

Declaración del investigador/persona que realiza el procedimiento de consentimiento informado:

He leído con precisión la hoja de información al/la potencial participante y me aseguré lo mejor que pude que el/la participante comprende lo que implica este estudio. Confirmo que el/la participante tuvo la oportunidad de hacer preguntas sobre el estudio y todas las preguntas formuladas fueron respondidas correctamente lo mejor que pude; el/la participante no fue forzado/a a dar su consentimiento, que se dio de forma libre y voluntaria; y se le ha entregado una copia de este formulario.

**Persona que realiza el procedimiento de consentimiento informado:**	
**Nombre y apellido**	
**Firma**	
**Fecha**	

ANEXO 3: FICHA DE VISITA BASAL

**Código de investigación de participante:**		**PE - _ _ _**	
**Fecha de registro de datos:** _ _/_ _ _/_ _ _ _		**Hora:** _ _: _ _ (24 horas)	
**INFORMACIÓN DEMOGRÁFICA**			
**Apellido Paterno**	**Apellido Materno**	**Nombres**	
**Fecha de nacimiento**	**Edad**	**Sexo**	**Procedencia**
_ _ /_ _ _ /_ _ _ _	______ **años**	☐ **M** ☐ **F**	_______________
**Teléfono de paciente**	**Historia Clínica N**		**Documento de Identidad**
**Domicilio**		**Distrito**	**Establecimiento de Salud**
**Nombre de familiar reponsable / soporte**			**Teléfono familiar**

Llenar los datos y marcar con un "X" en la casilla que corresponda

**ANTECEDENTES PERSONALES**			
¿Fuma o consume tabaco? ☐Sí ☐No		¿Cuántos cigarrillos fuma al día?	
¿Toma Alcohol? ☐ Sí ☐ No		¿Qué cantidad de bebidas consume por semana?	
**Diagnóstico**	**Fecha de inicio**	**Fecha de inicio**	**¿Continúa?**
**Inmunosupresión**	☐Si ☐No	_ _/_ _ _/_ _ _ _	☐Sí ☐No
**EPOC**	☐Si ☐No	_ _/_ _ _/_ _ _ _	☐Sí ☐No
**Diabetes mellitus (Tipo I o II)**	☐Si ☐No	_ _/_ _ _/_ _ _ _	☐Sí ☐No
**Hipertensión arterial**	☐Si ☐No	_ _/_ _ _/_ _ _ _	☐Sí ☐No
**Enfermedades hepáticas** ………………………………………….	☐Si ☐No	_ _/_ _ _/_ _ _ _	☐Sí ☐No
**VIH**	☐Si ☐No	_ _/_ _ _/_ _ _ _	☐Sí ☐No
**Insuficiencia renal**	☐Si ☐No	_ _/_ _ _/_ _ _ _	☐Sí ☐No
**Enfermedad coronaria**	☐Si ☐No	_ _/_ _ _/_ _ _ _	☐Sí ☐No
**Otros** *(Especficar):* ……………………………	☐Si ☐No	_ _/_ _ _/_ _ _ _	☐Sí ☐No

EVALUACIÓN MÉDICA BASAL	
**SIGNOS VITALES**	
Frecuencia respiratoria __ __ __ por minuto	Presión arterial __ __ __ / __ __ __ mmHg
Temperatura (oral) __ __.__ °C	Saturación de oxígeno __.__%
Peso __ __ __. __ Kg	Talla __ __ __.__ cm
IMC __ __. __ Kg/m^2	Tiempo de enfermedad : __ __ días
**EVALUACIÓN DE SINTOMAS Y SIGNOS**	
¿Presenta el paciente algún signo o síntoma anormal en el examen físico?	☐Sí ☐No

EXAMEN FÍSICO

Signos y síntomas		Presente	Grado (1-4)	Signos y síntomas	Presente	Grado (1-4)
**General**				**Músculoesquelético**		
1.	Fiebre reportada	☐Sí ☐No		17.Dolor de espalda	☐Sí ☐No	
2.	Malestar general	☐Sí ☐No		18.Lesión de tendon	☐Sí ☐No	
3.	Fatiga	☐Sí ☐No		19.Artralgia	☐Sí ☐No	
**Respiratorio**				20.Mialgia	☐Sí ☐No	
4.	Tos	☐Sí ☐No		21.Otros	☐Sí ☐No	
5.	Dísnea	☐Sí ☐No		**Linfático**		
6.	Broncospasmo	☐Sí ☐No		22. Agrandamiento o sensibilidad de los ganglios	☐Sí ☐No	
7.	Anosmia	☐Sí ☐No		**Dermatológica**		
**Cardiovascular**				23. Prurito	☐Sí ☐No	
8.	Dolor de pecho	☐Sí ☐No		24.Erupción cutánea	☐Sí ☐No	
9.	Palpitaciones	☐Sí ☐No		25. Mucositis/ Estomatitis	☐Sí ☐No	
10.	Arritmias	☐Sí ☐No		**Neurológico.**		
11.	Edema	☐Sí ☐No		26. Cefalea	☐Sí ☐No	
12.	Síncope	☐Sí ☐No		27. Mareo	☐Sí ☐No	
**Gastrointestinal**				28. Convulsiones	☐Sí ☐No	
13.	Odinofagia	☐Sí ☐No		29.Letargo	☐Sí ☐No	
14.	Náusea	☐Sí ☐No		30.Confusión	☐Sí ☐No	
15.	Vómito	☐Sí ☐No		Oftalmológicas		
16.	Diarrea	☐Sí ☐No		31. Alteración visual	☐Sí ☐No	

HISOPADO NASOFARÍNGEO PARA SARS-COV-2		
**¿Cuál es el resultado de la prueba?**	☐Positivo	☐Negativo
**Fecha de resultado**	_ _ /_ _ _ /_ _ _	
**EXAMEN DE HECES (PRIMERA VISITA HASTA EL DÍA SIGUIENTE)**		
**¿Cuál es el resultado de la prueba?**	☐Presencia de Helmintos	☐Ausencia de Helmintos
**Fecha de resultado**	_ _ /_ _ _ /_ _ _	

ADMINISTRACIÓN DE PRODUCTO EN INVESTIGACIÓN

PRIMERA DOSIS		Entrada y fecha
¿Tomó el participante el producto en investigación?<br><br>☐Sí ☐No	**Fecha y hora**<br><br>_ _ /_ _ _ /_ _ _	

SEGUNDA DOSIS		Entrada y fecha
¿Tomó el participante el producto en investigación?<br><br>☐Sí ☐No	**Fecha y hora**<br><br>_ _ /_ _ _ /_ _ _	

TERCERA DOSIS		Entrada y fecha
¿Tomó el participante el producto en investigación?<br><br>☐Sí ☐No	**Fecha y hora**<br><br>_ _ /_ _ _ /_ _ _	

ANEXO 4: FICHA MEDICACIÓN CONCOMITANTE

MEDICAMENTOS									
**Medicamento** [1]	**Unidad**	**Frecuencia**	**Dosis**	**Indicación** [2]	**Vía** [3]	**Fecha de inicio** *(dd/mes/aaaa)*	Marcar si continúa al final del estudio	**Fecha de finalización o en curso** [4] (dd/mes/aaaa)	**Entrada del personal** [5]
1.						__/__/____	☐	__/__/____ ☐ En curso	
2.						__/__/____	☐	__/__/____ ☐ En curso	
3.						__/__/____	☐	__/__/____ ☐ En curso	
4.						__/__/____	☐	__/__/____ ☐ En curso	
5.						__/__/____	☐	__/__/____ ☐ En curso	
6.						__/__/____	☐	__/__/____ ☐ En curso	
7.						__/__/____	☐	__/__/____ ☐ En curso	
8.						__/__/____	☐	__/__/____ ☐ En curso	
9.						__/__/____	☐	__/__/____ ☐ En curso	
10.						__/__/____	☐	__/__/____ ☐ En curso	

ANEXO 5: FICHA DE EVENTOS ADVERSOS

Listar todos los eventos adversos. Se debe hacer seguimiento a todos los EA hasta su resolución o hasta que se alcance un criterio de valoración clínica estable.

Evento adverso	Fecha de inicio<br>*dd/mes/aaaa*	Hora de inicio<br>1 = hora desconocida	Severidad<br>1 = Leve<br>2 = Moderada<br>3 = Severa<br>4 = Pone en riesgo la vida<br>5 = Muerte	Lo Esperado<br>1 – Esperado<br>2 - No se espera<br>3 - N / A	Relación con el medicamento de estudio<br>1 = No relacionado<br>2 = Probablemente no relacionado<br>3 = Posiblemente	Acción tomada<br>0 = Ninguna<br>1 = Medicamento<br>2 = Terapia no medicamentosa<br>3 = Hospitalización o prolongación<br>4 = Interrupción del medicamento de	Fecha de finalización<br>*dd/mes/aaaa*	Desenlace<br>1 = Se resolvió<br>2 = Se resolvió con secuelas<br>3 = En curso<br>4 = Crónico/esta	EAS<br>1=S1<br>2= No	Entrada del personal
1.	___/___/____						___/___/____			
2.	___/___/____						___/___/____			
3.	___/___/____						___/___/____			
4.	___/___/____						___/___/____			
5.	___/___/____						___/___/____			
6.	___/___/____						___/___/____			
7.	___/___/____						___/___/____			
8.	___/___/____						___/___/____			
9.	___/___/____						___/___/____			
10.	___/___/____						___/___/____			

ANEXO 6: FICHA DE VISITA SEGUIMIENTO

**Código de investigación de participante:**		**PE - _ _ _**							
**EVALUACIÓN DE SINTOMAS Y SIGNOS**									
**SIGNOS VITALES**		**4**		**7**		**14**		**21**	
Temperatura (oral) __ __.__ °C									
Frecuencia cardíaca __ __ __ por minuto									
Frecuencia respiratoria __ __ __ por minuto									
Presión arterial __ __ __ / __ __ __ mmHg									
Saturación de oxígeno __.__%									
**General**		**Sí**	**Grado (1-4)**	**Sí**	**Grado (1-4)**	**Sí**	**Grado (1-4)**	**Sí**	**Grado (1-4)**
1.	Fiebre reportada								
2.	Malestar general								
3.	Fatiga								
**Respiratorio**									
4.	Tos								
5.	Dísnea								
6.	Broncospasmo								
7.	Anosmia								
**Cardiovascular**									
8.	Dolor de pecho								
9.	Palpitaciones								
10.	Arritmias								
11.	Edema								
12.	Síncope								
**Gastrointestinal**									
13.	Odinofagia								
14.	Náusea								
15.	Vómito								
16.	Diarrea								

Código de investigación de participante:									PE - _ _ _
**VISITA DE DÍA**		**4**		**7**		**14**		**21**	
**Músculoesquelético**		**Sí**	**Grado (1-4)**	**Sí**	**Grado (1-4)**	**Sí**	**Grado (1-4)**	**Sí**	**Grado (1-4)**
17.	Dolor de espalda								
18.	Lesión de tendón								
19.	Artralgia								
20.	Mialgia								
21.	Otros								
**Linfático**									
22.	Agrandamiento o sensibilidad de los ganglios								
**Dermatológica**									
23.	Prurito								
24.	Erupción cutánea								
25.	Mucositits / Estomatitis								
**Neurológico**									
26.	Cefalea								
27.	Mareo								
28.	Convulsiones								
29	Letargo								
30.	Confusión								
**Oftalmológico**									
31.	Alteración visual								
**Laboratorio**		**4**		**7**		**14**		**21**	
		**Sí**	**Hora**	**Sí**	**Hora**	**Sí**	**Hora**	**Sí**	**Hora**
32.	Colección de muestras de sangre								
33.	Hisopado nasofaríngeo								
34.	Serología SARS-CoV-2 (pruebas rápidas)								
35.	Examen de heces (parásitos)								

ANEXO 7: FICHA DE EVALUACIÓN CLÍNICA DIARIA

Auto evaluación diaria de síntomas

Este cuestionario será un formulario electrónico, las respuestas son en su mayoría de selección simple. La columna "comentarios" es para uso interno. Los participantes sólo verán las preguntas y el panel de respuestas para seleccionar. La sección 12.1 del protocolo describe el proceso de almacenamiento.

Todas las preguntas se refieren a síntomas en las últimas 24 horas, o desde la última vez que cumplimentó en cuestionario. Sugerimos que lo complete cada día en la misma franja horaria			
	**Pregunta**	**Respuesta**	**Comentarios**
**Valoración global**	¿Cómo se encuentra usted hoy?	Mejor que ayer<br>Igual que ayer<br>Peor que ayer	
**Malestar general o cansancio**	¿Tiene malestar general o cansancio?	SI/NO	
	Su malestar o cansancio esta:	Mejor que ayer<br>Igual que ayer<br>Peor que ayer	En caso afirmativo
**Temperatura**	¿Se ha tomado la temperatura?	SI/NO	
	¿Ha tenido usted fiebre hoy?	SI/NO	
	¿Cuál ha sido su temperatura más alta desde ayer?	XX ◦C	En caso afirmativo
**Tos**	¿Tiene usted tos?	SI/NO	
	Su tos es:	Con flemas<br>Sin flemas	En caso afirmativo
	Su expectoración:	Es blanquecina<br>Es amarillenta<br>Es verdosa<br>Tiene sangre	Si tiene flemas
	Su tos esta:	Mejor que ayer<br>Igual que ayer<br>Peor que ayer	En caso afirmativo
	Su tos, ¿le dificulta el comer, beber o conversar?	SI/NO	En caso afirmativo
**Anosmia**	¿Puede usted percibir bien los olores?	SI/NO	
**Disgeusia**	¿Tiene usted mal sabor de boca?	SI/NO	
**Cefalea**	¿Tiene usted dolor de cabeza?	SI/NO	
**Odinofagia**	¿Tiene dolor de garganta?	SI/NO	
**Congestión nasal**	¿Tiene congestión nasal?	SI/NO	
**Disnea**	¿Tiene usted fatiga? (¿le falte el aliento?)	SI/NO	
	Se fatiga usted:	En reposo □<br>Al hablar □<br>Al andar □<br>Al subir	Todas las que apliquen

		escaleras □	
**Vómitos**	¿Tiene vómitos?	SI/NO	
	¿Son causados por la tos?	SI/NO	En caso afirmativo
	¿Cuántas veces ha vomitado desde ayer?	#	En caso afirmativo
**Diarrea**	¿Tiene diarrea?	SI/NO	
	¿Cuántas deposiciones ha tenido desde ayer?	SI/NO	En caso afirmativo
**Medicación**	¿Ha tomado usted alguna medicación para estos síntomas en las desde ayer?	SI/NO	
	En caso afirmativo, ¿Cuál?	*Texto libre*	

**Posibles efectos adversos de la ivermectina**	¿Desde ayer, ha experimentado alguno de estos síntomas?	Confusión/dificultad para concentrarse en algo □<br>Mareos □<br>Somnolencia □<br>Vértigo □<br>Temblores□	Todas las que apliquen
	¿Desde ayer, ha experimentado alguno de estos síntomas?	Visión borrosa □<br>Dificultad para enfocar objetos o para leer □<br>Visión de túnel □<br>Colores o formas anormales □<br>Puntos ciegos □<br>Puntos flotantes □	Todas las que apliquen
	¿Desde ayer, ha experimentado alguno de estos síntomas?	Prurito (picor) □<br>Sarpullido o erupción □	Todas las que apliquen

ANEXO 8: FORMULARIO DE CONSENTIMIENTO INFORMADO PARA PARTICIPAR EN UN ESTUDIO CLÍNICO (V 2.0)

CONSENTIMIENTO INFORMADO PARA PARTICIPAR EN UN ESTUDIO DE INVESTIGACIÓN	
**Título:**	Ensayo Clínico aleatorizado de Fase IIa para comparar la efectividad de la ivermectina versus placebo en la negativización del PCR en pacientes en fase temprana de COVID-19
**Investigadores:**	Dra. Patricia García, Dr. Jesús Chacaltana Dr. Carlos Chaccour, Dr. César Cárcamo, Dra. Patricia León, Dr. Germán Málaga, Dr. Hansel Mundaca, Dr. César Ugarte
**Patrocinador:**	Universidad Peruana Cayetano Heredia
**Institucion:**	Hospital Nacional Cayetano Heredia y Hospital Daniel Alcides Carrión

INTRODUCCION

Se le está invitando a participar en una investigación clínica, dirigida por la Universidad Cayetano Heredia, que ha sido aprobada por el Comité Nacional Transitorio de Ética en Investigación, porque Ud. ha pasado la fase de pre-selección para este estudio.

Es importante que entienda la diferencia entre la atención regular que obtiene de su médico y la que está relacionada a este estudio. El fin de la atención médica es monitorizar y mejorar su salud. El fin de un estudio de investigación es recabar información. Su participación en este estudio es totalmente voluntaria, nadie lo puede obligar a participar; puede decidir participar ahora y cambiar de opinión posteriormente.

Si decide participar en este estudio, se le pedirá que firme este documento de consentimiento antes de que se realice cualquier actividad relacionada al estudio. Recibirá una copia de este documento.

¿Cuál es el propósito del estudio de investigación?

Al momento no existe un tratamiento específico aprobado contra el COVID-19. En este estudio de investigación queremos evaluar si la ivermectina realmente puede tener actividad contra el virus del COVID-19. Para esto algunos de los participantes al azar recibirán Ivermectina y otros recibirán solo un placebo. El placebo es un compuesto farmacológico que no contiene ivermectina, pero que al tomarla le será indistinguible en sabor y apariencia. Ni siquiera el médico que lo trata sabrá si se le dio ivermectina o placebo. A todos los pacientes se les hará un seguimiento diario, y visitas domiciliarias. También se tomarán pruebas de sangre y moleculares para ver el efecto si hay o no efecto de la ivermectina.

¿Cuántas personas participarán en este ensayo clínico?

Se reclutará hasta 236 participantes mayores de edad diagnosticados con COVID-19 leve y con menos de 96 horas de inicio de síntomas típicos (fiebre, tos, dolor de cabeza, etc.), que hayan pasado las pruebas en la pre-selección. Los pacientes serán reclutados al acudir al Hospital Cayetano Heredia y Hospital Daniel Alcides Carrión.

¿Por qué se me ha elegido?

Se le invita a participar en este estudio de investigación debido a que usted presenta un cuadro clínico sospechoso de de COVID-19. Además, porque Ud. es mayor de 18 años, ha tenido síntomas de COVID-19 por menos de 96 horas, y que no ha tomado ivermectina anteriormente en cualquiera de sus presentaciones, y ha pasado las pruebas en la fase de pre-selección.

¿Cuánto tiempo durará mi participación en el estudio?

La duración de su participación en el estudio será de 21 días. Iniciará con el examen aquí en hospital, y lo seguiremos monitoreando y se le visitará en su domicilio o donde se encuentre en los días 4, 7, 14, 21.

¿Qué involucra el estudio?

Si Ud. decide participar, luego de haber respondido todas sus preguntas y haber firmado este consentimiento. Se completará la recolección de datos demográficos, antecedentes de salud, historia de la enfermedad y se le hará el

examen físico. Luego se le hará la toma de la muestra de su nariz y garganta usando un hisopo para confirmar la presencia del virus con la prueba molecular, le tomaremos una muestra de sangre (aproximadamente una cucharada) para unos estudios de anticuerpos (para ver cómo se defiende del virus). Además, debido a que se conoce que la presencia de parásitos intestinales podría influenciar la respuesta de las defensas de su cuerpo ante una infección, como por ejemplo el COVID, le pediremos una muestra de heces para descartar la presencia de parásitos. Si no pudiese dejar hoy una muestra de heces, le entregaremos un frasco para muestra de heces y mañana lo visitaremos para recogerlo.

Luego, se procederá a indicarle el tratamiento que le será asignado al azar. Ni usted ni el médico del estudio sabrá el tratamiento que recibirá, le podrá tocar el placebo o la ivermectina.

Basados en su peso, se le indicará una cantidad exacta de gotas correspondientes, y se le administrará por vía oral la primera dosis durante la primera visita bajo la supervisión directa del médico. Antes de retirarse, se le entregarán dos frascos adicionales correspondientes a la segunda y tercera dosis. Usted tomará estas dosis en su domicilio bajo monitoreo telefónico por el médico de estudio, el segundo y tercer día. Todos los días uno de los médicos del estudio se estará comunicando con Ud. por teléfono para preguntar como está y recoger información sobre sus síntomas, y si fuera el caso que presentará algún signo de alarma poder ayudarlo a que acuda al hospital y reciba el manejo requerido. Se le informará sobre su resultado de la prueba molecular tomada el primer día, y si esta es negativa allí termina su participación en el estudio.

Si su prueba molecular salió positiva, además de las llamadas se le visitará en casa los días 4, 7, 14 y 21 después de la primera visita. En estas visitas el médico del estudio le realizará un examen físico, tomará muestras de sangre para para pruebas rápidas de serología COVID-19, y tomará muestras de su nariz para hacer pruebas moleculares para el virus y se le tomará una muestra de sangre (aproximadamente una cucharada) para el estudio de anticuerpos. Adicionalmente, durante la visita a domicilio del día 14 se le colectará una segunda muestra de heces.

En caso de que el resultado basal de la prueba molecular sea positivo, se realizará cultivo viral de aquella y las subsecuentes muestras a tomar con la finalidad de evaluar la viabilidad del virus.

¿Cuáles son riesgos y potenciales beneficios del estudio?

La ivermectina es un medicamento muy seguro, usado por más de 2.700 millones de personas desde hace 30 años, sin embargo, pueden producir efectos secundarios temporales. Cuando se usa en pacientes infectados con el parásito intestinal *Strongyloides*, puede causar cansancio, dolor abdominal, disminución del apetito, diarrea, nausea, vómitos mareos, vértigo, temblor, prurito y erupciones, dolor de cabeza (todos en menos de 3% de los pacientes). Rara vez puede ocasionar alteraciones en análisis clínicos como aumento transitorio de enzimas hepáticas o reducción transitoria las células blancas. En el raro evento de una reacción alérgica, usted podría presentar erupción, picor, inflamación en labios o lengua o dificultad para respirar. Si esto ocurriese debe usted acudir de inmediato a urgencias y llevar una copia de este documento consigo.

La extracción de sangre tiene un pequeño riesgo de infección y puede ser un poco dolorosa. Su brazo podría tener algún hematoma durante algunos días.

Puede que el fármaco no tenga actividad contra el virus y que no haya ningún beneficio directo para usted por participar en el ensayo. Es posible que su participación nos ayude a responder a la pregunta de investigación sobre el potencial efecto de la ivermectina.

El equipo de estudio necesita saber si le ocurre algo inusual. Si usted se encuentra peor durante el tiempo que transcurra entre las visitas, por favor háganoslo saber, no es necesario esperar hasta la próxima visita.

Si es ingresado en el hospital durante el período de tiempo que dure el ensayo, por favor dígale al médico responsable de su asistencia que está participando en este estudio y pídale que se ponga en contacto con nosotros.

Medicamento alternativo

Actualmente no existe un tratamiento seguro y efectivo para manejar la infección por SARS-CoV-2. Los medicamentos disponibles no han sido probados, sin embargo, se permitirán medidas de apoyo al paciente, como el uso de antipiréticos e hidratación. Es un criterio de exclusión el estar tomando medicamentos como la quinidina, amiodarona, diltiazem, espironolactona, verapamilo, claritromicina, eritromicina, itraconazol, ketoconazol, ciclosporina, tacrolimus, indinavir, ritonavir, cobicistat o Warfarina que pueden interactuar mal con la ivermectina. Usted puede continuar tomando cualquier fármaco y terapia no farmacológica previa consulta con el médico del estudio.

Costos y pago

No hay costos por su participación en el estudio. Usted no tendrá que pagar por ningún medicamento ni por ninguna prueba del estudio. Usted no recibirá pago por participar en el estudio.

Confidencialidad

Usted será identificado con un código. Garantizamos mantener su privacidad de toda su información tal y como lo exige la ley. Su información médica y del estudio podrá ser revisada solo por el equipo del estudio, el CNTEI, y el Instituto Nacional de Salud del Perú. Los investigadores del centro se asegurarán de que cualquier persona que no esté relacionada con el estudio no tenga acceso a sus datos del estudio.

El embarazo y la lactancia

Debido a que se desconoce la seguridad de la ivermectina durante el embarazo y la lactancia, se le excluirá del ensayo si está embarazada o lactando.

Derechos de los participantes

Al dar su consentimiento para participar en este ensayo clínico, usted no renuncia a ninguno de sus derechos legales. Dar consentimiento significa que usted ha escuchado o leído la información sobre este ensayo clínico y que está de acuerdo en participar. Usted recibirá el formato original para sus registros personales. Si en algún momento usted se retira del ensayo clínico no sufrirá penalización alguna ni perderá ningún beneficio al que tiene derecho. Toda información este momento continuará siendo parte del estudio. Le daremos a conocer cualquier información nueva que pueda afectar su salud, bienestar o voluntad de permanecer en este estudio. Si usted quiere conocer los resultados del estudio, hágaselo saber al personal del estudio.

Compensación de lesiones relacionadas a investigación

Si usted sufre un daño o lesión relacionado directamente con el estudio, se le derivará para un tratamiento inmediato y gratuito a través del SIS, usted no tendrá que pagar por esta atención. No existe ningún otro programa de compensación. Usted no renuncia a ninguno de sus derechos legales al firmar este formulario de consentimiento.

PROBLEMAS O PREGUNTAS

Este estudio ha sido revisado y aprobado por el Comité Nacional Transitorio de Ética en Investigación para la evaluación y supervisión éticas de los Ensayos Clínicos de la enfermedad COVID-19 (CNTEI-COVID-19) para asegurarse de que los participantes de la investigación estén protegidos. Si usted tiene preguntas sobre sus derechos como participante, puede comunicarse al correo electrónico: comite_etica_covid19@ins.gob.pe y/o con el Dr. Aldo Vivar, Presidente del CNTEICovid-19, al teléfono: 992705648.

a) Contactos para responder cualquier duda o pregunta y en caso de lesiones - Investigadora Principal:
Dra. Patricia García por correo a patricia.garcia@upch.pe, o investigador Dr. Hansel Mundaca al teléfono 989 675 386 o escribiendo al correo electrónico hansel.mundaca@upch.pe.

b) Datos de contacto de la Autoridad Reguladora (INS). "Cuando usted considere que sus derechos son vulnerados o ante cualquier denuncia, usted puede contactarse con el INS (Oficina General de Investigación y Transferencia Tecnológica, OGITT), entidad reguladora de ensayos clínicos, a través del siguiente teléfono: 7481111 anexo 2191 o mediante comunicación escrita a través del siguiente correo electrónico: consultaensayos@ins.gob. pe, o mediante un documento formal presentado a través de mesa de partes de la institución o acudir en persona a la OGITT en la siguiente dirección: Cápac Yupanqui 1400, Jesús María, Lima 11".

Consentimiento Informado para participar en Estudio de Investigación

TÍTULO DEL ESTUDIO: Ensayo Clínico aleatorizado de Fase IIa para comparar la efectividad de la ivermectina versus placebo en la negativización del PCR en pacientes en fase temprana de COVID-19

Código de Pre-selección<br>Código de Ensayo clínico:	
Leí la información, o me fue leída. Pude hacer cualquier pregunta que quisiera, y recibí respuestas satisfactorias a mis preguntas	
	Doy mi consentimiento para participar en este estudio.
**Nombre y apellido**	
**Firma**	
**Fecha**	

Declaración del investigador/persona que realiza el procedimiento de consentimiento informado:

He leído con precisión la hoja de información al/la potencial participante y me aseguré lo mejor que pude que el/la participante comprende lo que implica este estudio. Confirmo que el/la participante tuvo la oportunidad de hacer preguntas sobre el estudio y todas las preguntas formuladas fueron respondidas correctamente lo mejor que pude; el/la participante no fue forzado/a a dar su consentimiento, que se dio de forma libre y voluntaria; y se le ha entregado una copia de este formulario.

**Persona que realiza el procedimiento de consentimiento informado:**	
**Nombre y apellido**	
**Firma**	
**Fecha**	

ANEXO 9: FORMULARIO DE CONSENTIMIENTO INFORMADO AUTORIZACIÓN DE ALMACENAMIENTO PARA USO FUTURO DE MUESTRAS (V2.0)

CONSENTIMIENTO INFORMADO PARA ALMACENAMIENTO DE MUESTRAS PARA USO FUTURO	
**Título:**	Ensayo Clínico aleatorizado de Fase IIa para comparar la efectividad de la ivermectina versus placebo en la negativización del PCR en pacientes en fase temprana de COVID-19
**Investigadores:**	Dra. Patricia García, Dr. Jesús Chacaltana, Dr. Carlos Chaccour, Dr. César Cárcamo, Dra. Patricia León, Dr. Germán Málaga, Dr. Hansel Mundaca, Dr. César Ugarte
**Patrocinador:**	Universidad Peruana Cayetano Heredia
**Institución:**	Hospital Nacional Cayetano Heredia y Hospital Daniel Alcides Carrión

Sobre el formato de consentimiento informado

Usted ha decidido participar en la investigación clínica, dirigida por la Universidad Cayetano Heredia, que evalúa el potencial de ivermectina para disminuir el porcentaje de paciente con una prueba PCR tomada de secreciones nasales al día 7. Ahora en este consentimiento informado le estamos preguntando si usted desea dar su permiso para que sus muestras de sangre sean congeladas y almacenadas en el laboratorio del Hospital Nacional Cayetano Heredia para uso futuro, cuando se consiga el financiamiento para hacerlas en nuestro laboratorio.

Estas muestras se almacenarán localmente en el Laboratorio de Enfermedades Tropicales del Hospital Nacional Cayetano Heredia con congeladores para almacenar las muestras, y se usarán para investigación relacionada a la inmunidad. Cualquier uso de su información y de sus muestras para otro estudio deberá ser aprobado por un comité de ética de nuestro país.

¿Con qué frecuencia se almacenarán mis muestras y qué pasará con ellas?

Se obtendrán y almacenarán cuatro muestras de sangre, de 10ml cada una, para estudios posteriores de inmunología y otros relacionados a COVID. La extracción de sangre se llevará a cabo en la visita basal (hoy) y en días 7, 14 y 21.

No se realizarán pruebas genéticas con estas muestras. Las muestras pueden ser almacenadas por un período máximo de 5 años, incluso una vez terminado el estudio. Estas muestras serán usadas solo para fines de entender mejor el sistema inmune (el sistema de defensas del cuerpo) frente a la infección por COVID-19.

¿Qué pruebas se realizarán con mis muestras?

Los estudios inmunológicos serán:

- Células inmunes innatas (células dendríticas plasmacitoides y mieloides, células NK, macrófagos pro-inflamatorios, intermedios y clásicos) medidas en PBMC crio-preservado por flujo de citometría.
- Células CD4+ T y CD8+ T (% total de CD4+T y CD8+ T) expresando cualquier marcador funcional dentro de la simulación in vitro de PBMC con péptidos SARS-CoV-2, medidos por flujo de citometría.

La muestra de sangre se conservará a -20C hasta por 5 años. Se usará el código de investigación de participante para mantener la confidencialidad. No se realizarán estudios genéticos con las muestras de sangre almacenadas.

¿Dónde se almacenarán mis muestras?

En el caso que usted esté de acuerdo y nos da su permiso mediante este formato, sus muestras serán almacenadas en el:

-Laboratorio de Enfermedades Tropicales del Hospital Nacional Cayetano Heredia
Av. Honorio Delgado nº430 urb. Ingeniería San Martín de Porres,
Lima – Perú

¿Qué sucede si ahora estoy de acuerdo en que mis muestras se almacenen y después cambio de opinión?

Las personas tienen el derecho a dejar de participar en una investigación o decidir que ya no quiere que se almacene sus muestras, usted puede tomar contacto con el personal de la sede. Ellos le comunicarán con el responsable del Laboratorio de Enfermedades Tropicales del Hospital Nacional Cayetano Heredia, para comunicarles que no se deben estudiar y que estas se deben destruir.

En caso de no aceptar este almacenamiento no se verá afectada su participación en el estudio

¿Cuáles son los riesgos que tengo al aceptar que se almacenen mis muestras?

Esta información de las muestras se toma como parte de este estudio y no hay riesgos adicionales por almacenarlas. Sus muestras están almacenadas con un número de código de modo que no hay riesgos de pérdida de su privacidad.

¿Qué hay acerca de la confidencialidad?

Para proteger su confidencialidad no almacenaremos sus muestras con ninguna información que pudiera identificarlo(a) a usted. Colocaremos una etiqueta a todas sus muestras con un código en lugar de usar su nombre (con los protectores usuales de la identidad).

¿Tengo que pagar algo?

Usted no tiene que pagar por el almacenamiento de sus muestras.

PROBLEMAS O PREGUNTAS

Este estudio ha sido revisado y aprobado por el Comité Nacional Transitorio de Ética en Investigación para la evaluación y supervisión éticas de los Ensayos Clínicos de la enfermedad COVID-19 (CNTEI-COVID-19) para asegurarse de que los participantes de la investigación estén protegidos. Si usted tiene preguntas sobre sus derechos como participante, puede comunicarse al correo electrónico: comite_etica_covid19@ins.gob.pe y/o con el Dr. Aldo Vivar, Presidente del CNTEICovid-19, al teléfono: 992705648.

a) Contactos para responder cualquier duda o pregunta y en caso de lesiones **-** Investigadora Principal:
Dra. Patricia García por correo a patricia.garcia@upch.pe, o investigador Dr. Hansel Mundaca al teléfono 989 675 386 o escribiendo al correo electrónico hansel.mundaca@upch.pe.

b) Datos de contacto de la Autoridad Reguladora (INS). "Cuando usted considere que sus derechos son vulnerados o ante cualquier denuncia, usted puede contactarse con el INS (Oficina General de Investigación y Transferencia Tecnológica, OGITT), entidad reguladora de ensayos clínicos, a través del siguiente teléfono: 7481111 anexo 2191 o mediante comunicación escrita a través del siguiente correo electrónico: consultaensayos@ins.gob.pe, o mediante un documento formal presentado a través de mesa de partes de la institución o acudir en persona a la OGITT en la siguiente dirección: Cápac Yupanqui 1400, Jesús María, Lima 11".

Consentimiento Informado para Almacenamiento de Muestras para Uso Futuro (V 2.0)

TÍTULO DEL ESTUDIO: Ensayo Clínico aleatorizado de Fase IIa para comparar la efectividad de la ivermectina versus placebo en la negativización del PCR en pacientes en fase temprana de COVID-19

Código de Pre-selección<br>Código de Ensayo clínico:	
Leí la información, o me fue leída. Pude hacer cualquier pregunta que quisiera, y recibí respuestas satisfactorias a mis preguntas	
	Autorizo que se guarden mis muestras de sangre para estudios posteriores |sobre COVID-19
**Nombre y apellido**	
**Firma**	
**Fecha**	

Declaración del investigador/persona que realiza el procedimiento de consentimiento informado:

He leído con precisión la hoja de información al/la potencial participante y me aseguré lo mejor que pude que el/la participante comprende lo que implica este estudio. Confirmo que el/la participante tuvo la oportunidad de hacer preguntas sobre el estudio y todas las preguntas formuladas fueron respondidas correctamente lo mejor que pude; el/la participante no fue forzado/a a dar su consentimiento, que se dio de forma libre y voluntaria; y se le ha entregado una copia de este formulario.

**Persona que realiza el procedimiento de consentimiento informado:**	
**Nombre y apellido**	
**Firma**	
**Fecha**	
